# Humanin activates integrin αV–TGFβ axis and leads to glioblastoma progression

**DOI:** 10.1038/s41419-024-06790-8

**Published:** 2024-06-28

**Authors:** Cuong P. Ha, Tuyen N. M. Hua, Vu. T. A. Vo, Jiyeon Om, Sangwon Han, Seung-Kuy Cha, Kyu-Sang Park, Yangsik Jeong

**Affiliations:** 1 Department of Biochemistry, Wonju College of Medicine, Yonsei University, Wonju, 26426 Republic of Korea; 2 Department of Global Medical Science, Wonju College of Medicine, Yonsei University, Wonju, 26426 Republic of Korea; 3 Mitohormesis Research Center, Wonju College of Medicine, Yonsei University, Wonju, 26426 Republic of Korea; 4 Department of Ophthalmology, Wonju College of Medicine, Yonsei University, Wonju, 26426 Republic of Korea; 5 Department of Physiology, Wonju College of Medicine, Yonsei University, Wonju, 26426 Republic of Korea; 6 Institutes of Lifestyle Medicine, Wonju College of Medicine, Yonsei University, Wonju, 26426 Republic of Korea; 7 Mitochondrial Medicine, Wonju College of Medicine, Yonsei University, Wonju, 26426 Republic of Korea; 8 Present Address: Department of Pharmacology - Clinical Pharmacy, Faculty of Pharmacy, Pham Ngoc Thach University of Medicine, Ho Chi Minh City, Vietnam

**Keywords:** Cancer stem cells, Tumour angiogenesis, Cancer metabolism

## Abstract

The role of mitochondria peptides in the spreading of glioblastoma remains poorly understood. In this study, we investigated the mechanism underlying intracranial glioblastoma progression. Our findings demonstrate that the mitochondria-derived peptide, humanin, plays a significant role in enhancing glioblastoma progression through the intratumoral activation of the integrin alpha V (ITGAV)–TGF beta (TGFβ) signaling axis. In glioblastoma tissues, humanin showed a significant upregulation in the tumor area compared to the corresponding normal region. Utilizing multiple in vitro pharmacological and genetic approaches, we observed that humanin activates the ITGAV pathway, leading to cellular attachment and filopodia formation. This process aids the subsequent migration and invasion of attached glioblastoma cells through intracellular TGFβR signaling activation. In addition, our in vivo orthotopic glioblastoma model provides further support for the pro-tumoral function of humanin. We observed a correlation between poor survival and aggressive invasiveness in the humanin-treated group, with noticeable tumor protrusions and induced angiogenesis compared to the control. Intriguingly, the in vivo effect of humanin on glioblastoma was significantly reduced by the treatment of TGFBR1 inhibitor. To strengthen these findings, public database analysis revealed a significant association between genes in the ITGAV–TGFβR axis and poor prognosis in glioblastoma patients. These results collectively highlight humanin as a pro-tumoral factor, making it a promising biological target for treating glioblastoma.

## Introduction

Glioblastoma, one of the most aggressive types of tumors, leads to extremely poor quality of the patients’ life and prognosis, with 15 months of a median and 5.5% of a 5-year survival rate [1–3]. This deadly grade IV astrocytoma mostly accompanies tumor relapse with acquired drug resistance, which may be attributable to intratumoral heterogeneity potentially mediated by phenotypic cellular transitions and/or intratumoral communication between glioblastoma subtypes [4–8]. Originating from the subventricular zone in the brain [9], a single tumor-initiating clone continues to proliferate into a highly heterogeneous glioblastoma tumor by acquiring further genetic and/or biological alterations and spreads throughout the brain.

Glioblastoma is characterized by an intricate interplay of factors that collectively contribute to its invasive nature. Included are various factors, TGF-β for tumor invasion via epithelial-to-mesenchymal transition (EMT) as well as immunosuppressive microenvironment [10, 11], MMPs and Cathepsins for extracellular matrix (ECM) remodeling to aid invasion and angiogenesis [12].

Glioblastoma progression is also closely associated with mitochondrial dysfunction, particularly with metabolism alteration and genetic abnormality in mitochondrial complexes associated with ROS generation [13–17]. A recent study reported that mitochondria are directly transferred between patient-derived glioblastoma stem cells (GSCs) through tunneling nanotubes, and the transfer increases upon irradiation [18]. While intra- and inter-cellular mitochondrial dysfunction might play a pivotal role in glioblastoma progression, little is known about the mitochondrial factors involved in its intracranial spread [19, 20].

Humanin is a mitochondria-derived functional peptide (MDP) known for neuronal protection from β-amyloid and cytoprotection through interaction with insulin-like growth factor binding protein 3 (IGFBP3) and Bax [21–23]. A part of the mitochondrial *16s* ribosomal RNA encodes this secreted 21 or 24 amino acid peptide depending on where it is translated, and is sometimes further modified with formylation at the N-terminal end of the peptide [21, 24]. Circulating humanin may function through the cell surface receptors, FPRL1, or the trimeric complex CNTFR/WSX-1/gp130 [25]. Further studies have reported that various functional humanin-derivatives and nuclear-encoded orthologs show similar or enhanced activity [26, 27]. Humanin’s cytoprotective role may result in unwanted effects in many cancers. In fact, compared with healthy donors, humanin is highly expressed in triple-negative breast cancer (TNBC) biopsies [28]. Furthermore, exogenous administration of the humanin peptide reduced the cancer cell sensitivity to doxorubicin in TNBC-bearing mice [28]. Likewise, humanin knockdown increased tumor susceptibility to apoptotic stimuli in pituitary tumors and glioblastoma models [22, 29]. In another report, Agudelo et al. proposed that humanin analog could reduce the sensitivity of glioblastoma against chemotherapy [30]. Although it is associated with pro-tumoral function, humanin’s mechanism of action in glioblastoma still remains elusive. We hypothesized that since astrocytes can release humanin [31] and the abnormal proliferation of astrocytes progresses to glioblastoma, humanin might be involved in this progression.

In the present study, we found that humanin, as a potential ligand for integrin receptors, induced the adherence of glioblastoma stem cells (GSCs) via cytoskeleton remodeling through integrin receptor activation and further cell migration triggered by released TGFβ. Humanin-mediated activation of the integrin–TGFβ axis contributes to enhanced invasiveness of GSCs, eventually leading to a more aggressive phenotype of glioblastoma. Taken together, these results provide insights into how humanin can drive glioblastoma pathogenesis, which can be exploited for further therapeutic strategies.

## Results

### Humanin is expressed in the brain

**Fig. 1:**
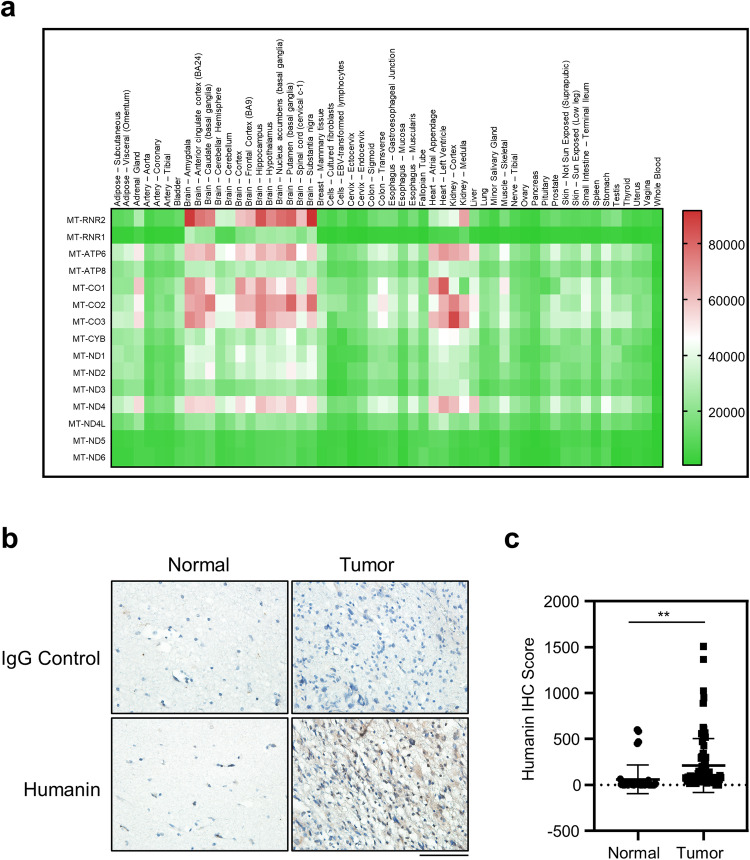
Humanin is upregulated in GBM tumors. **a** Humanin is highly expressed in brain tissues. Public GTEx portal resources were utilized for heatmap analysis of mitochondrial gene expression in 54 micro-dissected tissues from healthy donors. Included are *MT-RNR1* and *-RNR2* encoding 12s rRNA and 16s rRNA, respectively; *MT-ATP6* and *-ATP8* as ATP synthase membrane subunits for complex V; *MT-CO1*, *-CO2*, and *-CO3* for cytochrome c oxidases or complex IV; *MT-CYB* for cytochrome b; *MT-ND1–ND6* for NADH:Ubiquinone oxidoreductase core subunits or complex I. Note that TPM, transcripts per million, values for genes of interest are color-coded, red for high and green for low expression. **b**, **c** Humanin expression is higher in the glioblastoma tumor tissue compared to the pair-matched normal tissue. Using tissues from the glioblastoma patients, IHCs were performed for the expression of humanin peptide (**b**), followed by quantification (**c**). Each dot in the quantification graphs represents an individual staining region. Data information: In **c** statistical analysis was performed using a 2-tailed Student’s *t*-test. **P < 0.01. Scale bar 100 μm.

To explore the physiological relevance of humanin in the brain, we first analyzed public datasets, including the Genotype-Tissue Expression (GTEx) resource for the mitochondrial transcriptome. From the GTEx analysis, we found a subset of mitochondrial genes highly expressed in most brain regions but relatively decreased in the cerebellar hemisphere, cerebellum, heart, and kidneys. Mostly located in the heavy strand of mitochondria genome are the gene sets including *MT-ATP6*, ATP synthase membrane subunit 6 (complex V), *MT-CO1*, *-CO2*, *-CO3* as components of cytochrome c oxidase (complex IV), *MT-ND4*, NADH dehydrogenase 4 (complex I), and *MT-RNR2* that encodes the humanin peptide in the *16s* ribosomal RNA. In particular, the humanin peptide exhibited a distinguishable expression pattern in most areas of the brain, kidney medulla, and whole blood (Fig. 1a, Supplementary Fig. 1a). Using the same dataset, further analysis of nuclear-encoded humanin-like peptides revealed that *MT-RNR2L8* and *MT-RNR2L12*, encoding humanin-like peptide 8 (HL8) and 12 (HL12), respectively, showed the same expression pattern as humanin, while the overall expression level was considerably lower than that of humanin (Supplementary Fig. 1b). Although at different chromosomal locations, *HL8* and *HL12* encode identical amino acid sequences but Ser replacing Leu at position 12 in humanin peptide [32]. We further investigated whether humanin expression was pathologically associated with glioblastoma tumors. Using eight pairs of normal and corresponding glioblastoma tissues, we performed immunostaining for humanin expression, which exhibited significantly higher expression in the tumor area compared with the corresponding normal tissue of the same patients (Fig. 1b, c, Supplementary Fig. 1c, d). These results suggested that mitochondria-derived humanin is highly expressed in the brain and is pathologically relevant to glioblastoma pathogenesis.

### Humanin induces cellular attachment in GSCs

**Fig. 2:**
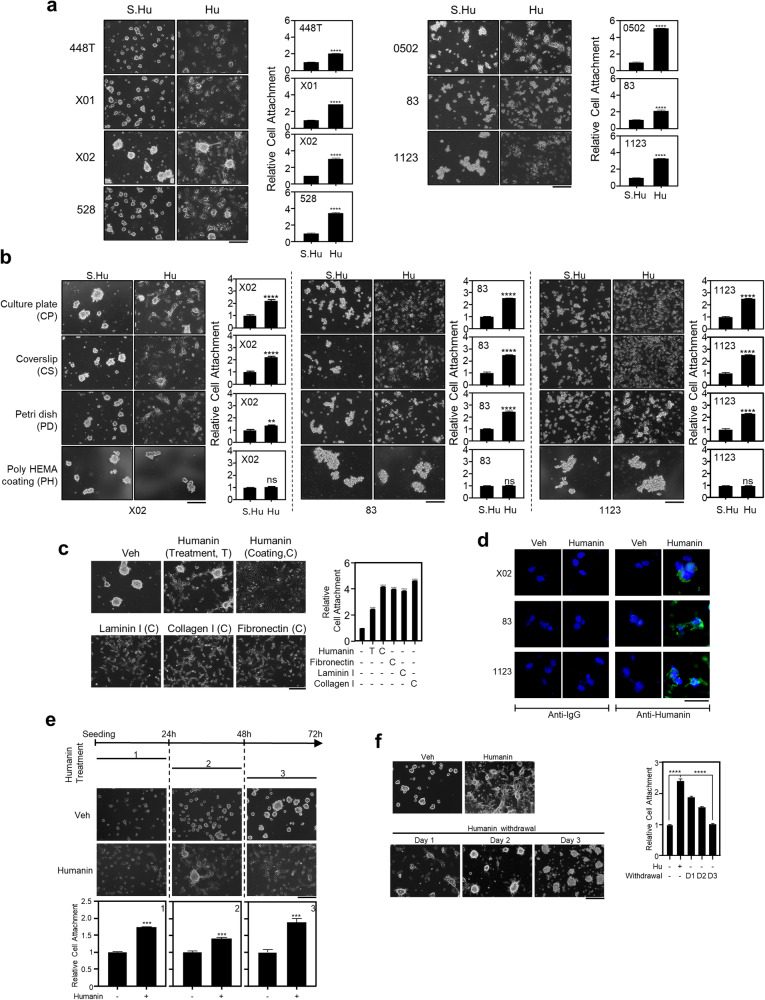
Humanin induces GSCs attachment. **a**, **b** GSCs were treated with 20 μM of scrambled humanin (S.Hu) or humanin (Hu) for 24 h in normal culture plate (**a**) or different culture surfaces, (**b**) including coverslip, Petri dish, poly-HEMA coating followed by observation of morphological changes using brightfield microscope (left) or quantification for cell attachment assay (right). **c** X02 cells were directly treated with 20 μM humanin or cultured on the plates after coating with humanin or other ECM proteins for 24 h, followed by observation of morphological changes using a brightfield microscope (left) or quantification for cell attachment assay (right). T treatment, C coating. **d** Humanin binds to the cell surface. Represented is a set of confocal microscope images for humanin binding after GSC treated with 20 µM humanin. **e** The extent of X02 sphere formation is not associated with humanin-induced attachment. X02 cells were pre-incubated for 0, 24, and 48 h, followed by 20 µM humanin treatment for 24 h. Images for morphological change (middle) and quantification (bottom) are represented. **f** Humanin-induced attachment is reversible. X02 cells were treated with 20 µM of humanin for 24 h, and subsequently, cellular attachment of X02 cells was assayed at 24, 48, and 72 h after withdrawal of humanin. Data information: In **a**, **b**, **e** statistical analysis performed using a two-tailed Student’s *t*-test. ***P < 0.001, ****P < 0.0001. In **c**, **f** statistical analysis was performed using one-way ANOVA. ***P < 0.001, ****P < 0.0001. Scale bar 250 μm or 20 μm (**d**) for images. Each experiment was performed with 3 replicates.

To understand the biological functions of humanin, we examined various cellular responses to humanin treatment in a panel of GSCs, including 448T, X01, X02, 528, 0502, 83, and 1123 cells. Humanin treatment induced the adherence of all suspended GSCs in a time-dependent manner, whereas HL8/HL12 did not show such effect (Fig. 2a, Supplementary Fig. 2a, b). The combination of humanin and HL8 showed a stronger effect on cellular attachment than individual treatments, suggesting that humanin and HL8 could be functionally complexed for the additive attachment effect (Supplementary Fig. 2b). However, under the same treatment conditions, neither attachment nor any morphological change was shown in the suspended SCLC cells (Supplementary Fig. 2c). These results imply that cellular attachment is a specific humanin-mediated event in GSCs. We further analyzed humanin-mediated GSC attachment under multiple biophysical conditions, including culture plate (CP), coverslip (CS), Petri dish (PD), and poly-HEMA (PH) hydrogel coating, which are generally known to inhibit cellular attachment [33]. Humanin was still able to attach GSCs to coverslips and Petri dishes, but not to the poly-HEMA coating (Fig. 2b). This suggested that humanin alone, regardless of any chemical coating, is sufficient to induce GSC attachment. In addition, humanin-mediated GSC attachment was comparable to that of other known biological inducers, including laminin, collagen I, and fibronectin (Fig. 2c). In fact, immunostaining showed that humanin binds to the cell surface of GSCs (Fig. 2d). Further characterization revealed that humanin adhered GSCs to the plate surface regardless of their sphere-formation ability (Fig. 2e). However, humanin withdrawal completely prevented the GSCs from adhering to the suspension state (Fig. 2f). Consistent with this finding, humanin treatment did not induce the expression of genes involved in the cellular attachment (Supplementary Fig. 2d). Taken together, these results suggested that unknown, humanin-related mediators function specifically on the surface of GSCs.

### Humanin-induced attachment depends on integrin

**Fig. 3:**
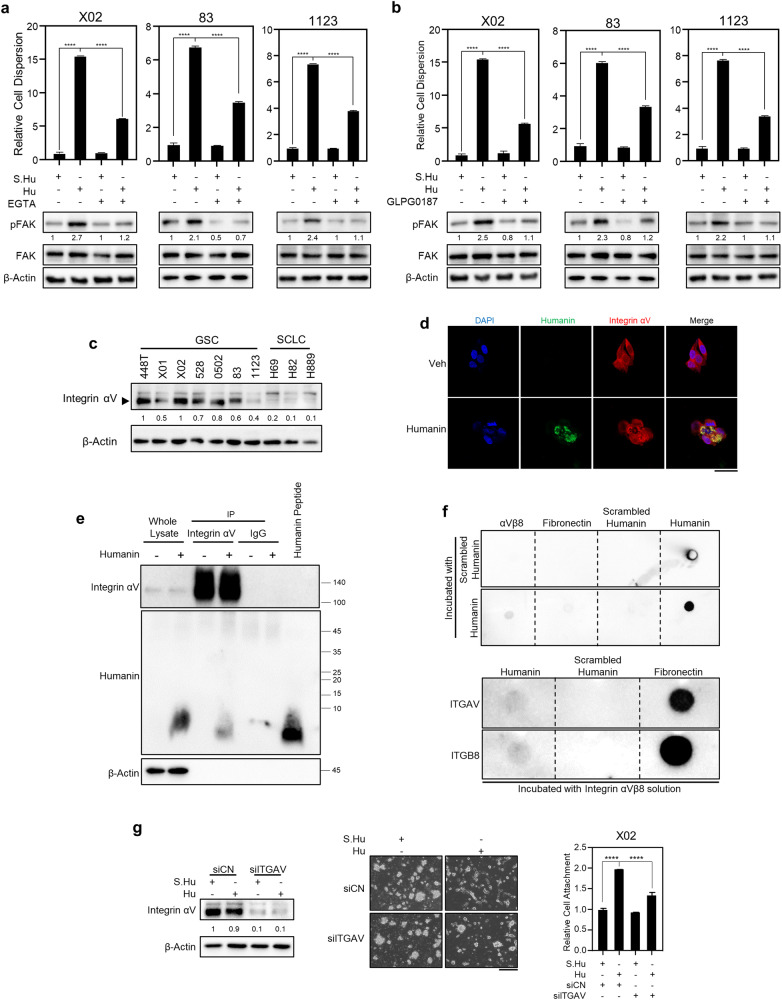
Humanin-induced cellular dispersion of the GSCs depends on integrin αV. **a**, **b** Inhibition of integrin αV reverses the humanin-induced dispersion of the GSCs. X02, 83, and 1123 cells were treated with 20 μM of scrambled humanin or humanin in combination with integrins inhibitors, 2 mM of EGTA (**a**), or 10 µM of GLPG0187 (**b**), for 24 h followed by quantification of cellular dispersion after attachment (upper) and immunoblot assay for phosphorylation of FAK (lower). **c** Basal expression of integrin αV in GSCs and SCLCs. **d**–**f** Humanin directly binds to integrin αVβ8. **d** Co-immunostaining of humanin and integrin αV. X02 cells were treated with 20 µM humanin for 24 h and followed by co-immunofluorescence assay for humanin and integrin αV. **e** Co-immunoprecipitation assay of integrin αV and humanin peptide. X02 cells were treated with 20 µM humanin peptide for 24 h before collecting cell lysate for co-immunoprecipitation assay. **f** Dot-blot assays were executed between humanin and other proteins, including fibronectin, scrambled humanin, or humanin. Indicated peptides, αVβ8, fibronectin, scrambled humanin, or humanin, were dotted onto a nitrocellulose membrane followed by incubating with humanin or scrambled humanin solution for interaction assay. Immunoblot assay was performed using humanin antibody (upper). Humanin, scrambled humanin, or fibronectin peptide was dotted onto nitrocellulose membrane, followed by the incubation with integrin αVβ8 solutions. Immunoblot assay was performed using integrin αV or β8 antibody (lower). **g** X02 cells were treated with 20 μM of scrambled humanin or humanin for 24 h, with or without integrin αV knockdown followed by immunoblot assay (left), cell attachment (middle), or morphological observation using a brightfield microscope (right). Data information: Every western data was quantified using densitometry. In **a**, **b**, **g** Statistical analysis was performed using one-way ANOVA; ****P < 0.0001. Scale bar 20 μm (**d**) or 250 μm (**g**) for images.

To investigate the molecular mechanisms by which humanin attaches to suspended GSCs, we performed multiple biological assays. First, as multiple proteins were previously reported to have potential binding to humanin [34], we examined the expression difference of the binding partners in GSCs vs. SCLCs showing no attachment to plate by humanin. Included are IGFBP3 and BCL2 for direct interaction and WSX-1, GP130, CNTFR, and FPRL1 for potential humanin receptors (Supplementary Fig. 3a). The expression of conventional humanin-binding factors, or even downstream STAT3 and ERK, showed no distinguishable patterns of expression between the two types of cancer cells (Supplementary Fig. 3b). In addition, humanin treatment did not induce further activation of STAT3 or ERK (Supplementary Fig. 3c), indicating that humanin may function through an unknown humanin-binding factor. As integrins are known to facilitate cell-cell and cell-ECM adhesion [35], we hypothesized that humanin-induced GSC attachment in a manner similar to multiple integrin ligands such as collagen I, fibronectin, and laminin. Based on the calcium dependency of integrin-mediated attachment [36, 37], GSCs were treated with EGTA, an extracellular calcium chelator, to determine whether calcium is necessary for humanin-induced attachment. Indeed, humanin-induced cell attachment was significantly reversed by EGTA treatment to chelate extracellular divalent calcium and magnesium ions, which induced cell attachment in a dose-dependent manner (Fig. 3a, Supplementary Fig. 3d). Similarly, treatment with the integrin blocker GLPG0187 decreased the effect of humanin in the same GSC panel (Fig. 3b). Consistent with these results, FAK phosphorylation, which is a hallmark of integrin-mediated intracellular signaling activation [38], was increased by humanin treatment, but was suppressed when treated with EGTA or GLPG0187 (Fig. 3a, b, Supplementary Fig. 3e). Notably, EGTA-AM treatment for intracellular calcium chelation showed no inhibitory effect on the humanin-induced attachment or changes in integrin and TGFβ signaling (Supplementary Fig. 3f, g). This suggests that extracellular calcium is critical for humanin action on cellular attachment.

Next, we wondered which isoform of integrin was involved in the attachment process. A previous study suggested the role of integrin αV in the regulation of cell attachment, where the knockdown of integrin αV resulted in the loss of cell attachment [39]. Compared to SCLC cells, the expression of integrin αV was higher in GSCs, in which humanin showed the ability to induce attachment (Fig. 3c, Supplementary Fig. 3h). To examine whether humanin is a potential ligand for the integrin receptor, we performed several dot blot, co-immunoprecipitation, and co-immunostaining assays to evaluate the interaction between integrin αV and humanin. All assays consistently showed direct molecular interactions between humanin and integrin αV (Fig. 3d–f, Supplementary Fig. 3i). These results were further confirmed by a loss-of-function study showing reduced humanin-mediated attachment of X02 and 83 cell line when integrin αV was knocked down (Fig. 3g, Supplementary Fig. 3j). Taken together, these results identified integrin αV as a molecular mediator and potential receptor for humanin peptide on the cell surface for humanin-induced attachment of GSCs.

### Humanin facilitates cell attachment by mediating F-actin remodeling

**Fig. 4:**
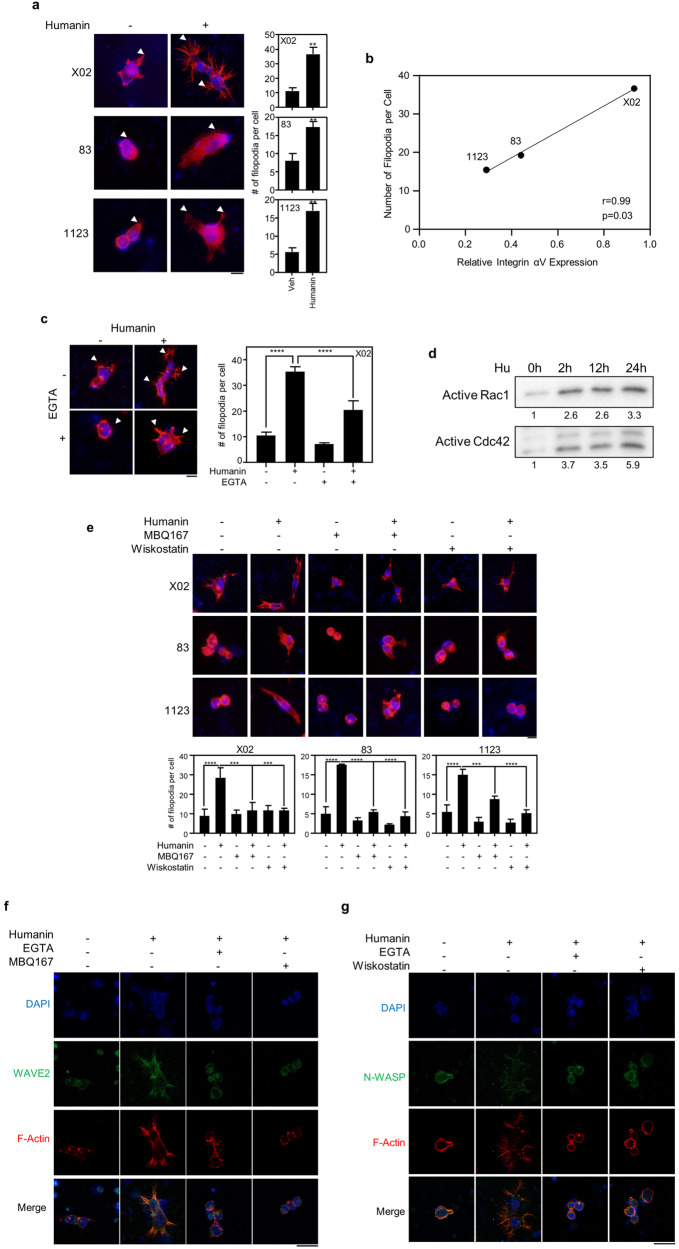
Humanin elicits filopodia formation by F-actin remodeling. **a** Representative images of filopodia formation upon 20 μM of humanin treatment for 24 h (left) and quantification for numbers of filopodia (right) in X02, 83, and 1123 cells. Arrowheads indicate filopodia in each cell line. **b** Correlation of integrin αV expression and the number of filopodia in GSCs. **c** Pharmacological evaluation of humanin-induced filopodia formation. Filopodia staining after 20 µM of humanin treatment in the presence of 2 mM EGTA (left) and quantification (right). **d** Immunoblots for the activation of Rac1/Cdc42 pathway. X02 cells were treated with 20 μM of humanin time-dependently, ranging from 2 h to 24 h. **e** Pharmacological evaluation of humanin-induced filopodia formation. Filopodia staining after 20 μM of humanin treatment in the presence of 50 nM MBQ167 for Rac/Cdc42 inhibitor, or 5 µM Wiskostatin for N-WASP inhibitor for 24 h followed by filopodia number quantification (bottom). **f**, **g** Co-immunofluorescence image for humanin-induced filopodia formation. X02 cells were treated with 2 mM EGTA, 50 nM MBQ167, or 5 µM Wiskostatin in the presence or absence of 20 µM of humanin, followed by immunohistochemistry for F-actin and WAVE-2 (**f**) or N-WASP (**g**) proteins. Data information: Every western data was quantified using densitometry. In **a** statistical analysis performed using a two-tailed Student’s *t*-test. In **c**, **e** statistical analysis was performed using one-way ANOVA. *P < 0.05, **P < 0.01, ***P < 0.001, ****P < 0.0001. Scale bar 20 μm for images.

We determined whether integrin-mediated intracellular signaling is accompanied by cytoskeletal changes in GSCs upon humanin treatment. We found filopodia formation in GSCs probably being preceded by the humanin-induced attachment (Fig. 4a), which correlated with integrin αV expression levels (Fig. 4b). The number of filopodia per cell was significantly higher in the humanin treatment group than in the control group, in which the Cell-tak was coated before cell seeding (Fig. 4a). Furthermore, the extent of cellular dispersion was clearly extended upon humanin treatment, which may be associated with cell migration (Fig. 4a). Consistently, extracellular calcium chelation by EGTA treatment significantly reversed the humanin-induced filopodia formation (Fig. 4c). Considering that Rho-GTPase family members mediate the intracellular integrin signaling pathway [40], we pharmacologically examined RhoA, Cdc42, and Rac1 for their role in cytoskeletal changes potentially accompanying cellular motility. While a previous study reported changing the suspended melanoma into an adherent state [41], Y27632, a ROCK inhibitor, did not induce attachment of glioblastoma X02 cells (Supplementary Fig. 4). Interestingly, humanin treatment increased the expression of active form of Rac1/Cdc42, a critical indicator for the activity of this pathway (Fig. 3d). Furthermore, when the cells were treated with MBQ167, a common inhibitor of both Rac1 and Cdc42 signaling, humanin-induced filopodia formation was significantly reversed. Similarly, treatment with Wiskostatin, a specific inhibitor of Cdc42 downstream of N-WASP, revealed the same inhibition as MBQ167 (Fig. 4e). Furthermore, humanin-mediated filopodia formation is associated with F-actin remodeling. When treated with humanin, the intracellular signaling pathway for actin polymerization becomes activated while suppressed with EGTA, Wiskostatin, or MBQ167 treatment (Fig. 4f, g). These results suggested that both Rac1 and Cdc42, but not RhoA, are responsible for F-actin remodeling via intracellular signal transduction from humanin and integrin αV interactions.

### Humanin induces integrin–TGFβ crosstalk

**Fig. 5:**
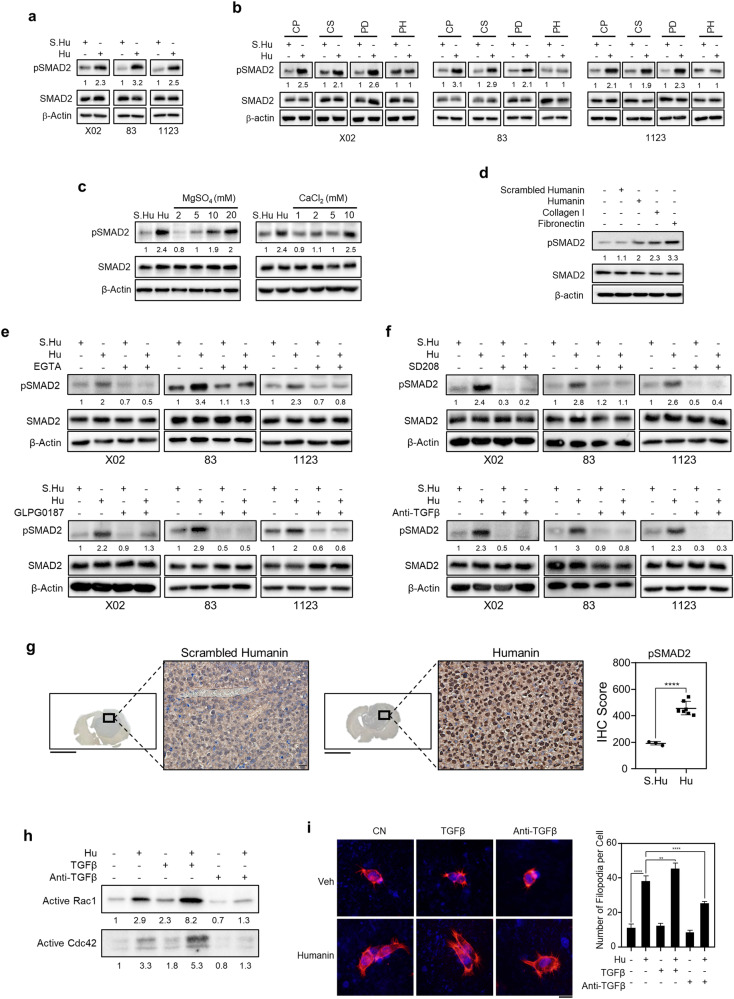
Humanin activates TGFβ signaling. **a**, **b** X02, 83, and 1123 cells were treated with 20 μM of scrambled humanin or humanin on culture plate (CP) (**a**) or with different culture surfaces (**b**) including coverslip (CS), Petri dish (PD), poly-HEMA (PH) coating for 24 h followed by immunoblot assay to evaluate the expression of pSmad2 and total Smad2. **c** X02 cells were treated with 20 μM of scrambled humanin or humanin or MgSO_4_ (left) and CaCl_2_ (right) in a dose-dependent manner for 24 h, followed by immunoblot assay to evaluate the expression of proteins of interest. **d** Immunoblot assay for TGFβ signaling activation in X02 cells upon treatment of 20 μM scrambled humanin or humanin, and in the condition of collagen I or fibronectin coating for 24 h. **e**, **f** Chemical or biological evaluation of the humanin-induced TGFβ signaling. X02, 83, and 1123 cells were treated with 20 μM of scrambled humanin or humanin in the presence of integrin inhibitors, including 2 mM of EGTA and 10 µM of GLPG0187 (**e**) or TGFβ signaling pathway inhibitors including 1 μg/mL anti-TGFβ antibody and 10 μM of SD208 (**f**) for 24 h, followed by immunoblot assay for proteins of interest. **g** Humanin induces phosphorylation of Smad2 in tumor tissues. IHCs for pSmad2 were executed in the xenografted tumors, showing representative images (left) and quantification (right). **h**, **i** Humanin-induced filopodia formation partly depends on TGFβ signaling. X02 cells were treated with 20 μM of humanin in the presence of 0.5 ng/mL TGFβ or 1 μg/mL anti-TGFβ for 24 h, followed by immunoblot for the active Rac1/Cdc42 expression (**h**), fluorescence images of filopodia formation (**i**, left) or quantification of filopodia (**i**, right). Scale bar 20 µm for images. Data information: Every western data was quantified using densitometry. In **g** statistical analysis was performed using a two-tailed Student’s *t*-test. ****P < 0.0001. In **i** one-way ANOVA. **P < 0.01, ****P < 0.0001. Scale bar 20 μm for images (**i**) or 1 cm for whole brain (**g**).

Since humanin-induced attachment is dependent on integrin αV, we next investigated whether humanin can also regulate integrin–TGFβ crosstalk. In fact, humanin treatment activated the canonical pathway of TGFβ signaling, indicated by increased pSmad2 within 24 h (Fig. 5a, Supplementary Fig. 5a), but not the non-Smad or non-canonical pathway of TGFβ signaling, such as p38 activation, which was suppressed by humanin [42] (Supplementary Fig. 5b). Smad2 phosphorylation in 448T cells was observed in a time-dependent manner upon humanin treatment (Supplementary Fig. 5a, lower). Smad2 phosphorylation, representing TGFβ signaling activation, was attenuated with the poly-HEMA coating surface (PH) but not under other conditions, which prevented cell attachment (Figs. 2b, 5b), suggesting that cell adhesion was a prerequisite for activation of the TGFβ signaling pathway. Consistently, divalent cations, including Mg^2+^ and Ca^2+^ or various ECM molecules that activated integrin and attachment in a similar way as humanin treatment (Figs. 2c, 3a, Supplementary Fig. 3d), increased Smad2 phosphorylation (Fig. 5c, d) in the GSC panel, but not in H82 cells showing non-attached features of SCLCs upon humanin treatment (Supplementary Figs. 2c, 5c). We further confirmed the relay of the humanin–integrin–TGF axis using multiple biological loss-of-function assays. These include EGTA treatment to chelate divalent ions, to neutralize TGFβ signaling by treatment with anti-TGFβ antibody and SD208 treatment to block TGFβ receptor activation, and GLPG0187 treatment to inhibit the integrin αV receptor (Fig. 5e, f). Humanin-induced pSmad2 expression was markedly attenuated by EGTA and GLPG0187 co-treatment (Fig. 5e), suggesting that humanin-induced TGFβ activation was dependent on integrin. In contrast, treatment with the TGFβ receptor inhibitor SD208 or TGFβ antibody attenuated humanin-induced pSmad2 but did not affect cell attachment, unlike integrin receptor inhibitors (Fig. 5f, Supplementary Fig. 5e). Notably, humanin-induced Smad2 phosphorylation was markedly inhibited by GLPG0187 but not by cilengitide specifically inhibiting αVβ3 and αVβ5, suggesting that the integrin αVβ6 or αVβ8 complex might be involved in humanin function [43, 44] (Supplementary Fig. 5d). The increase in Smad2 phosphorylation was further confirmed in xenografted tumor tissues upon humanin administration (Fig. 5g). These results suggest that TGFβ signaling activation is a secondary effector necessary for tumor progression after cell attachment induced by integrin activation. Indeed, GSCs remained in a suspended state with highly upregulated pSmad2 (Supplementary Fig. 5f) while showing partial changes in filopodia formation as well as the relevant molecular signaling upon treatment with TGFβ or anti-TGFβ antibody (Fig. 5h, i). Taken together, these results indicated that humanin induces integrin for attachment, subsequently followed by canonical TGFβ signaling pathway through integrin–TGFβ crosstalk.

### Humanin enhances tumor progression

**Fig. 6:**
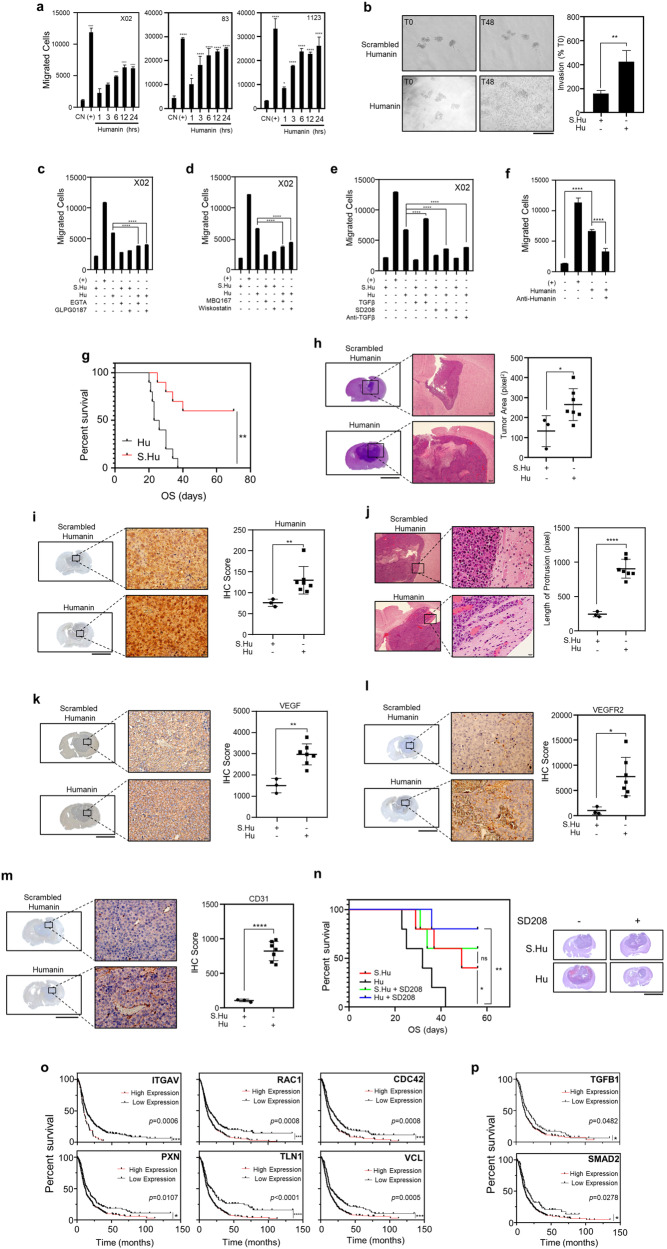
Humanin elicits cell migration of the GSCs. **a**–**f** Evaluation of humanin-induced cell migration of the GSCs. X02, 83, and 1123 cells were treated with 20 μM humanin in a time-dependent manner followed by 3D invasion or cell migration assay (**a**). Invasiveness of 83 cells was assayed upon humanin or scrambled humanin treatment. The invasion was represented in the picture (left) with quantification by using ImageJ software (right) (**b**). X02 cells were treated with 20 μM of scrambled humanin or humanin in the presence of 2 mM EGTA, 10 μM GLPG0187 for integrins inhibitors (**c**), 5 µM Wiskostatin for N-WASP inhibitor or 50 nM MBQ167 for Rac/CDC42 inhibitor (**d**), or 0.5 ng/mL TGFβ, 1 µg/mL anti-TGFβ, or 10 µM SD208 for TGFβ receptor (**e**) for 24 h, followed by cell migration assay. **f** Inhibition of humanin-induced migration by the anti-humanin antibody. X02 cells were treated with 20 μM of humanin in the presence or absence of 5 μg/mL anti-humanin antibody for 24 h, followed by cell migration assay. Data information: In **a**, **c**–**f** Statistical analysis was performed using one-way ANOVA. Statistical analysis was performed using one-way ANOVA. *P < 0.05, **P < 0.01, ***P < 0.001, ****P < 0.0001. In **b** two-tailed Student’s *t*-test. **P < 0.01. Scale bar 250 μm for images. Each experiment was performed with 3 replicates. **g** In vivo evaluation of humanin in a stereotaxic tumor model. Using the orthotopic xenograft tumor model of the 83 GSC, Kaplan–Meier’s plot is presented for survival analysis in the scrambled humanin (*n* = 10) or humanin-treated (*n* = 10) groups. **h**, **i** H&E staining and IHC analysis for the xenografted tumor tissues. H&E staining (left) for the xenografted tumor with relative tumor size (right) (**h**); IHC for humanin in the xenografted tumor tissues (**i**). IHCs for humanin were executed in the xenografted tumors, showing representative images (left) and quantification (right). **j**–**m** Humanin leads to tumor invasiveness. Tumor protrusion was represented in H&E staining (left) with quantification (right) (**j**); Humanin induces angiogenesis (**k**–**m**). Protein expression relevant to angiogenesis was examined in the xenografted tumor tissues. Included are VEGF (**k**), VEGF receptor 2 (**l**), and CD31 (**m**) staining. **n** Therapeutic evaluation using in vivo stereotaxic tumor model. Using the orthotopic xenograft tumor model of the 83 GSC, Kaplan–Meier’s plot is presented for survival analysis in the scrambled humanin or humanin-treated, with or without the treatment of SD208 (*n* = 5 in each group) (left). (Right) Representative images of tumor size in each group. Scale bar 1 cm. **o**, **p** Kaplan–Meier’s plot representing patient survival for genes associated with integrin–TGFβ axis. Using the Chinese Glioma Genome Atlas (CGGA) dataset, patient survival was analyzed for genes involved in the integrin (**o**) and TGFβ (**p**) signaling. Data information: In **h**–**m** statistical analysis was performed using a 2-tailed Student’s *t*-test. *P < 0.05, **P < 0.01, ***P < 0.001, ****P < 0.0001. In **n–p** log-rank test; *P < 0.05, **P < 0.01, ***P < 0.001, ****P < 0.0001.

We examined the biological invasiveness of GSCs using in vitro and in vivo systems to investigate the involvement of humanin-activated integrin–TGFβ axis in cancer hallmarks, such as invasion, metastasis, and angiogenesis, which are relevant to the patient’s prognosis and clinical outcome [45, 46]. Humanin-induced the migration of X02, 83, and 1123 GSCs as early as 3 h after treatment, which was further confirmed through in vitro invasion assay (Fig. 6a, b, Supplementary Fig. 6b). In turn, this migration was significantly reversed by blocking integrin signaling, either with EGTA or GLPG0187 to inhibit extracellular signaling (Fig. 6c, Supplementary Fig. 6a) or treatment with MBQ167 or Wiskostatin to inhibit intracellular WAVE/WASP signaling (Fig. 6d, Supplementary Fig. 6a). Similarly, direct inhibition of the TGFβ receptor by SD208 or anti-TGFβ antibody decreased the humanin effect, while treatment with TGFβ alone showed no change in cell migration, confirming that cell attachment is a prerequisite process for TGFβ-mediated cell migration in GSCs (Fig. 6e, Supplementary Fig. 6a). These observations were further supported by the live-cell imaging, which showed that humanin increased the relative travel distance and velocity. Conversely, treatments with integrins inhibitor GLPG0187 and anti-TGFβ significantly reduced these effects (Supplementary Fig. 6c, d, Supplementary Video 1). Notably, anti-humanin antibody treatment also showed a significant reduction in GSC migration (Fig. 6f, Supplementary Fig. 6a), suggesting that targeting functional humanin could be a potential candidate for glioblastoma intervention.

Next, to assess the humanin role for glioblastoma progression in vivo, we established an orthotopic xenograft mouse model by intracranial injection of GSC 83 that were cultivated with humanin or scrambled humanin peptide as a control. The humanin peptide group exhibited significantly shorter survival compared to the control peptide, which was supported by visualizing the bigger tumor gross of the humanin group compared with the scrambled humanin group (Fig. 6g, h, Supplementary Fig. 6e). Surprisingly, we found that humanin staining was significantly higher in the humanin-treated tumor tissues compared with scramble-treated ones (Fig. 6i). Further IHCs revealed that humanin elicited higher invasiveness of tumors spreading into adjacent normal tissues, which is supported by induced blood vessel formation showing increased VEGF, VEGFR2, and CD31 expression in the humanin-treated tumor tissues (Fig. 6j–m, Supplementary Fig. 6f). In addition, to further strengthen our hypothesis, we performed an in vivo experiment combining scrambled humanin or humanin with the treatments of SD208, an inhibitor for TGFβ pathway. Consistent with our prior findings, humanin group showed lower survival as compared to the scrambled humanin group. Intriguingly, treatment with SD208 not only significantly reduced tumor size but also increased the survival of the mice, and this effect observed exclusively in the humanin group, but not the scrambled humanin group (Fig. 6n). In addition to the non-clinical assessment of the pathophysiological function of humanin, we further explored the humanin–integrin–TGFβ axis using public glioblastoma datasets. In fact, higher levels of integrin αV, Rac1, and Cdc42 as downstream mediators or Paxillin, Talin1, or Vinculin as a downstream effector were strongly associated with poor prognosis in glioblastoma patients (Fig. 6o). Likewise, the glioblastoma group with higher expression of TGFβ1 or Smad2 showed a poor prognosis compared with that with lower expression (Fig. 6p).

Overall, these results suggested that targeting humanin may be a potential therapeutic strategy for glioblastoma.

## Discussion

**Fig. 7:**
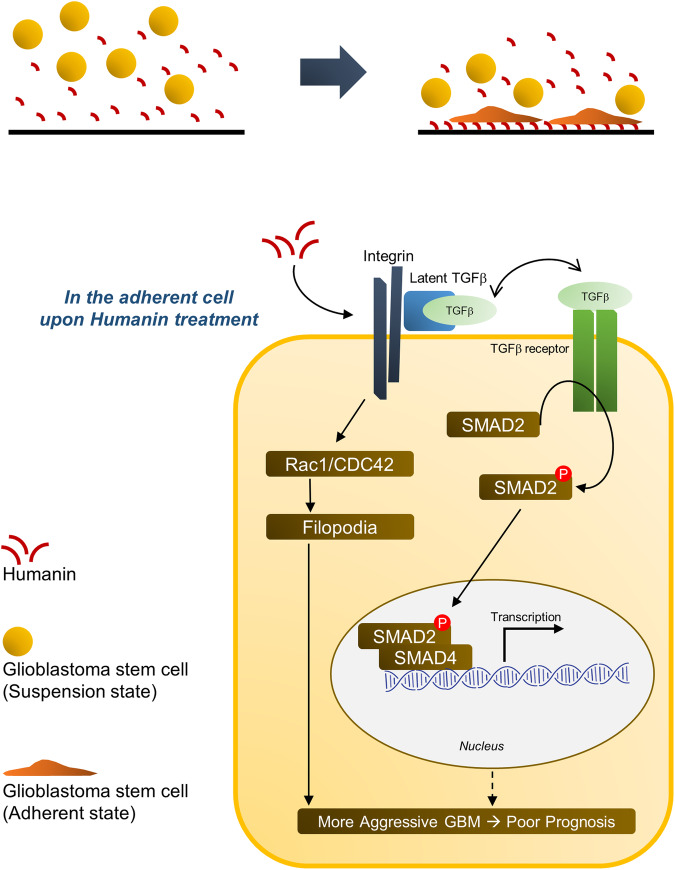
Hypothetical model of humanin function. Humanin treatment induces cell attachment in GSCs via integrin αV (upper). The activation of humanin-induced integrin signaling further triggered filopodia formation via Rac1/CDC42 and canonical TGFβ signaling pathway, which eventually supported cell migration and angiogenesis for the more aggressive GBM phenotype (lower).

Glioblastoma is one of the most aggressive cancer types and shows a devastating quality of life accompanied by an extremely poor prognosis in patients [47]. This fatal clinical outcome might be attributable to the intracranial spreading of the original tumor cells and glioblastoma heterogeneity, which could be attributed to intratumoral commensalism [8, 9, 48]. Although glioblastoma stem cells arise from the subventricular zone, the spread of initial cancer stem cells over the brain remains poorly understood [9]. Recent studies on intratumoral commensalism involve humoral factor dependency [8] and physical transfer of mitochondria through tunneling nanotubes between neighboring GSCs [18], suggesting that mitochondrial function is involved in glioblastoma progression. Here, we systematically demonstrated that the mitochondria-derived peptide humanin activates the integrin αV–TGFβ signaling axis, further enhancing glioblastoma progression by increasing tumor cell invasion and angiogenesis. To this end, we used multiple molecular and cell biological approaches to utilize in silico, in vitro, and in vivo systems. In brief, we found that (1) humanin remarkably induced GSC attachment in a similar way to ECM proteins; (2) mechanistically, humanin directly binds to and activates integrin αV relaying to the canonical TGFβ signaling pathway; and (3) biologically, the humanin–integrin αV–TGFβ axis triggered GSC migration, leading to the shorter survival of the orthotopic mouse model treated with humanin (Fig. 7). Pathologically, humanin induces intratumoral angiogenesis, which may contribute to the aggressiveness of glioblastoma.

Along with previous reports of various beneficial effects, including neuronal cytoprotective effects, anti-inflammatory response, and improved insulin sensitivity [49, 50], our findings provide a clue to explain how the mitochondrial peptide humanin contributes to intracranial tumor spreading, which includes consecutive events of cellular attachment, filopodia formation, and migration (Fig. 7). We believe that humanin-induced cellular attachment is dependent on the activation of integrin receptors but not on TGFβ signaling, as cellular attachment is still observed even upon blocking TGFβ signaling. As shown by complete or partial suppression by integrin inhibition or anti-TGF treatment, filopodia formation may be contributed by both signaling pathways, mainly intracellular integrin signaling and partly by TGFβ signaling. Finally, cell migration is mainly attributable to TGFβ signaling activation, as humanin-induced migration increases with exogenous TGFβ treatment. Although the present study proposes an interesting observation for humanin function in glioblastoma progression, it is important to have further discussion on some biological aspects for a couple of issues raised by this finding.

First, over the past two decades, humanin has been recognized as a beneficial peptide with properties that promote cell survival, reduce oxidative stress, and modulate inflammation across various conditions, including neurodegenerative diseases, cardiovascular health, diabetes, and aging [24, 51]. Indeed, several reports have demonstrated that humanin can be utilized as a therapeutic strategy. Notably, Chai et al. demonstrated that treatment with humanin in rats enhanced long-term potentiation and mitigates memory deficits induced by Aβ_1–42_ in Alzheimer’s models [52]. Additionally, a recent report by Yen et al. illustrated that the injection of HNG, a potent humanin analog, effectively improved age-related cognitive symptoms in vivo [53].

One of our intriguing observations is that, humanin appears to exhibit considerable stability, as evidenced by its detection in the brain by IHC staining several weeks after the initial injection. Given the relatively low physiological levels of endogenous humanin, we hypothesize that these signals originated from the exogenous sources of humanin. However, we cannot overlook the possibility that humanin treatment may also trigger the production of endogenous humanin within the cell as reported earlier [54]. The cytoprotective role of humanin, while beneficial in numerous scenarios, might have detrimental effects in cancer contexts. Our findings suggest that despite its advantages, it’s important to recognize that prolonged exposure to high concentrations of humanin could also potentially promote the initiation and progression of glioblastoma [30].

Second, nuclear-encoded HL8/HL12, unlike mitochondrial humanin, induced no attachment of GSCs. Having replaced leucine with serine at position 12 in the humanin peptide, HL8/HL12 completely lost its capability to induce integrin αV–TGFβ mediated cell attachment as well as migration of GSCs. This suggests that the humanin effect on cell attachment is unique and specific. Similarly, several previous studies have included human derivatives that function in various biological processes [21, 22, 55]. For example, the HNG peptide, substituted with serine by glycine at position 14, acquired stronger potency in various biological processes, including cell growth and cytoprotective effects in age-related macular degeneration, compared to humanin [56, 57]. Considering the current findings and previous reports, it would be worthwhile to explore humanin mimetics with therapeutic potential for glioblastoma.

Finally, the limitation of the experimental approach using exogenous humanin peptides may lie on its strong hydrophobic property due to mainly consisting of non-polar residues. The hydrophobic nature of humanin induces a high tendency to aggregate, which could be an explanation why micromolar levels are required to elicit biological activities [58, 59]. On the other hand, the serum concentration of humanin has been reported to be in the sub-nanomolar range [51, 60]. It is conceivable that humanin levels in neural tissues would be higher than those in serum; nevertheless, further investigations are required to demonstrate physiologic or pathophysiologic implications of endogenous humanin.

In summary, we described the pro-tumoral function of humanin in GBM through integrin αV activation, leading to filopodia formation and further TGFβ signaling activation for migration, which provides a novel mechanism of humanin action in cancer biology.

## Materials and methods

### Cell culture and reagents

The cells used in this manuscript were kindly provided by Prof. Jong Bae Park (National Cancer Center, South Korea) (448T, X01, X02, 528, 83, and 1123) and Prof. Myung-Jin Park (KIRAM, South Korea) (0502) [61]. GSCs were cultured in DMEM/F12 (Corning, Cat# 10-090-CV) with B27 supplements (Thermo Fisher Scientific, Cat# 17504044), EGF (R&D Systems, Cat# 236-EG-200), and bFGF (R&D Systems, 233-FB-025). Small-cell-lung-cancer (SCLCs), provided by Prof. John D. Minna from the University of Texas Southwestern Medical Center, Dallas, Texas, USA, were cultured in RMPI (Corning, Cat# 10-041-CV) with 10% FBS (Gibco, Cat# 16000044) supplemented with 50 U/mL penicillin, and 50 U/mL streptomycin (Gibco, Cat# 15140-122), at 37 °C, 5% CO_2_ atmosphere. Scrambled humanin, humanin, and its analog, HL8/HL12, were obtained from Anygen (Gwangju, South Korea, Cat# AGP-8245). Y27632 (Cat# 688000) and EGTA (Cat# 324626) were purchased from Sigma–Aldrich (St. Louis, MO, USA). GLPG0187 (Cat# 205842) was purchased from Medkoo Bioscience (Morrisville, NC, USA). MBQ167 (Cat# HY-112842) and Wiskostatin (Cat# B7666) were obtained from MedChemExpress (Monmouth Junction, NJ, USA) and APExBio (Houston, TX, USA), respectively. SD208 (Cat. # 7624) was obtained from Selleck Chemicals GmbH (Houston, TX, USA). Recombinant TGF−β (Cat# 240-B) and anti-TGF−β (Cat# MAB1835) antibodies for neutralization were purchased from R&D Systems (Minneapolis, MN, USA).

### Gene-expression analysis in the Genotype-Tissue Expression (GTEx) resource

The expression analysis data of mitochondria-encoded and multiple humanin-like genes were downloaded from the publicly available GTEx portal (www.gtexportal.org) [62]. The analysis was performed with normalized quantification or Transcripts Per Kilobase Million (TPM) values across the transcripts of each sample using FLAIR quantification. GraphPad Prism 8 was used for heatmap visualization and relative expression analysis.

### Culture surface treatment

Humanin (Anygen, Cat# AGP-8245) was prepared in water, and laminin (Sigma–Aldrich, Cat# L2020) and fibronectin (Thermo Fisher Scientific, Cat# PHE0023) were diluted in phosphate-buffered saline (PBS). Collagen I (Gibco, Cat# A1048301) was dissolved in 20 mM acetic acid, whereas Cell-tak (Corning, Cat# 354230) was prepared in 0.67 M at NaHCO_3_ pH 8. The culture plate surface was covered with a sufficient volume of coating solution and incubated for 30 min at room temperature (humanin), 37 °C for 20 min (laminin), 6 h (fibronectin), or 20 min at room temperature (Collagen I and Cell-tak, respectively). The plates were washed with PBS and air-dried prior to use. The final concentration of humanin and extracellular matrix (ECM) was 10 µg/mL. Poly 2-hydroxyethyl methacrylate (poly-HEMA; Sigma–Aldrich, Cat# P3932) was dissolved in ethanol 95% overnight at 37 °C to a final concentration of 20 mg/mL, followed by culture surface coating. The plate was dried for 5–10 min before cell seeding.

### Cell attachment assay

Cells were treated as indicated, followed by washing with PBS to remove any debris before adding the crystal violet solution. After 20 min of incubation at 4 °C, the culture plates were gently washed with tap water to remove any residual crystal violet and air-dried at room temperature overnight. Methanol was added to the plates to solubilize the dye, followed by OD measurement at 590 nm for quantification using a spectrophotometer (Biotek).

### Immunoblot assay

Cells were collected and prepared in RIPA buffer for further analysis using immunoblot assay, as reported previously [63]. Primary antibodies including β-Actin (SCBT, Cat# sc-69879), Integrin αV (Cat# 4711), focal adhesion kinase (FAK, Cat# 3285), pFAK (Cat# 8556), pSmad2 (Cat# 3108), Smad2 (Cat# 5339), p38 (Cat# 8690), p-p38 (Cat# 4511), pAkt (Cat# 9275), Akt (Cat# 9272), pERK (Cat# 9101), ERK (Cat# 9102), were all obtained from Cell Signaling Technology. Horseradish peroxidase (HRP)-conjugated anti-mouse IgG (Cat# A16066) and anti-rabbit IgG (Cat# G21234; Innovative Research, Invitrogen) were used as secondary antibodies. The images were captured using ChemiDoc XRS+ system (Bio-Rad). Densitometry calculations were performed using Bio-Rad Image Lab software version 6.0.1.

### Stereotaxic mouse model

Animal experiments were approved by the Institutional Animal Care and Use Committee (IACUC) of Yonsei Wonju College of Medicine. Twenty mice were randomly divided into two groups: Scrambled humanin (control) and humanin; ‘83’ were prepared in DMEM/F12 at a concentration of 1000 cells per µL and mixed with scrambled humanin or humanin (15 μg/mice) at a 1:1 ratio. A total of 2 µL of the mixture was injected intracranially into 5-week-old female Balb/c nude mice using a stereotaxic frame (Stoelting, IL, USA, Cat#STL-51500) at 1 mm posterior, 1 mm lateral to the bregma, at a depth of 2.5 mm. Body weight and mouse condition were monitored every two days. Mice were sacrificed when they showed severe weight loss or neurologic symptoms or at the end of the experiment after 10 weeks of injection. Mouse brains were harvested for further analysis using hematoxylin and eosin (H&E) and immunohistochemistry (IHC) staining.

For the in vivo SD208 treatment, mice were divided into four groups (*n* = 5 each) and injected with cells mixed with either scrambled humanin or humanin to initiate tumor formation, following a similar protocol as mentioned above. After 3 days of the initial injection, mice was then treated with daily dose of either vehicle, 1% (w/v) methylcellulose (Sigma, Cat# M7027), or SD208 (60 mg/kg), administered by oral gavage (Jeungdo, Cat# JD-S-126-5202) for a duration of 3 weeks [64, 65]. Body weight and the condition of the mice were monitored daily throughout the experiment. Mice were euthanized if they exhibited severe weight loss or neurological symptoms, or at the conclusion of the study after 10 weeks post-injection.

### Immunohistochemistry staining

Paraffin-embedded sections were provided by Wonju Severance Christian Hospital with the approval of the Committee of Institutional Review Board (approval number: CR318068). Before staining, the sections (including both human GBM and 83-injected mouse samples) were rehydrated using Neoclear and a series of decreasing alcohol concentrations. After the antigen retrieval step with boiled citrate buffer (pH 6.0), samples were blocked with 0.3% H_2_O_2_ for 30 min before staining, as previously described using VECTASTAIN Elite ABC HRP kit (Vector, Burlingame, CA, Cat# PK-6100) [66]. Primary antibodies, including anti-humanin (Neuromics, Cat# RA19000), anti-pSmad2 (SCBT, Cat# sc-11769), anti-VEGF (SCBT, Cat# sc-7269), anti-VEGFR2 (CST, Cat# 2479 S), anti-CD31 (SCBT, Cat# sc-376764) and anti-IgG rabbit (Vector Laboratories, Cat# I-1000-5), were used at a final concentration of 10 μg/mL. Staining signals were detected using the ImmPACT DAB Peroxidase (HRP) Substrate (Vector, Cat# SK-4105). Samples were then counterstained with hematoxylin (Vector, Cat# H3401) before mounting with Permanent Mounting Medium (Vector, Cat# H-5000-60). Images were captured with an Olympus DP73 brightfield microscope and quantified using Quantitative Pathology & Bioimage Analysis (QuPath software platform) [67].

### H&E staining

The 83-injected sections were carefully rehydrated using Neoclear and a series of decreasing alcohols. Samples were then covered with hematoxylin for 3 min, followed by a brief wash with 0.1% HCl. All samples were washed thoroughly with water for 5 min before staining with eosin (3 min). The sections were washed again with water before mounting with Permanent Mounting Medium (Vector, Cat# H-5000-60). All images were captured using an Olympus DP73 brightfield microscope. The tumor area and length of protrusion were measured using the ImageJ software.

### Immunofluorescence staining

GSCs were seeded on precoated coverslips using Cell-Tak (Cat #354230; Corning, NY, USA) at a final concentration of 14 µg/mL, following the manufacturer’s instructions. After washing with PBS and air-drying coverslips for 15 min at room temperature, GSCs were seeded at 1.5 × 10^5^ cells per well for 2 d, followed by 1 d of humanin treatment. Cells were washed with PBS and fixed with 4% paraformaldehyde for 15 min at room temperature. After permeabilization with PBS and 0.2% Triton-X (Sigma) for 1 h, samples were incubated with anti-humanin (Neuromics, Cat# RA19000), anti-WAVE-2 (CST, Cat# 3659), anti-N-WASP (SCBT, Cat# sc-271484), anti-integrin αV (SCBT, Cat# sc-9969 AF594; 1:100 ratio) overnight as primary antibodies. The coverslips were then washed and stained with anti-rabbit Alexa Fluor 488 (Cat #R37116; Invitrogen) at a 1:200 ratio as a secondary antibody. For Phalloidin staining, Alexa Flour 594 Phalloidin (Invitrogen, Cat#A12381) was used to visualize F-actin after 1-h incubation of cells following the manufacturer’s instructions. The samples were then mounted with Vectarshield DAPI solution (Vectar Laboratories, Cat#H-2000-10) onto the slides. Images were visualized and captured using an Olympus DP73 microscope. The number of filopodia was quantified using the FiloQuant plug-in for ImageJ software (LOCI, University of Wisconsin, USA), as previously described [68]. The number of filopodia per cell is reported as mean ± SEM (*n* = 3).

For CD31 immunofluorescence staining in paraffin-embedded orthotopic tumor model, samples were first carefully rehydrated using Neoclear and a series of decreasing alcohol concentrations. After the antigen retrieval step with boiled citrate buffer (pH 6.0), samples were blocked with 0.3% H_2_O_2_ for 30 min using VECTASTAIN Elite ABC HRP kit. Slides were then incubated with CD31 first antibody (SCBT, Cat# sc-376764) at 1:100 ratio overnight. The slides were then washed and stained with anti-mouse Alexa Fluor 594 (Cat #A11032; Invitrogen) at a 1:200 ratio as a secondary antibody for 1 h and mounted with Vectarshield DAPI solution (Vectar Laboratories, Cat#H-2000-10). Images were visualized and captured using an Olympus DP73 microscope and quantified using “Vessel Analysis” plug-in in ImageJ [69].

### Co-Immunoprecipitation (co-IP) assay

After 24 h treatment with 20 µM humanin, GBM cells were collected using co-IP lysis buffer (50 mM Tris-HCl, 150 mM NaCl, 0.5% NP-40, with the addition of 1% protease inhibitor – Sigma, Cat# P8340, and phosphatase inhibitor – Roche, Cat# 04906845001). Cell lysates were then incubated with integrin αV antibody at a 1:50 ratio (CST, Cat# 4711 S) overnight (4 °C) before adding Pierce Protein A/G agarose (Thermo Scientific, Cat# 20421) to pulldown the protein of interest (room temperature, 2 h). The solutions were then centrifuged (15000 rpm, 4 °C) to remove the supernatant. The remaining pellets were then washed four times with co-IP lysis buffer (10 min, room temperature) before adding blue loading buffer (CST, Cat# 7722 S). All the incubation and washing steps were performed on a rotating wheel. The samples were then subjected to western blotting against integrin αV (CST, Cat# 4711 S), humanin (Neuromics, Cat# RA19000), and β-actin (SCBT, Cat# sc-69879). Horseradish peroxidase HRP-conjugated anti-mouse IgG (Invitrogen, Cat# A16066) and anti-rabbit IgG (Invitrogen, Cat# G21234) were used as secondary antibodies. The membranes were detected using an ECL solution (GE Healthcare, Cat# RPN2235).

### Dot-blot assay

To identify the in vitro interaction between humanin and the proteins/peptides of interest, a dot-blot assay was carried out, as reported previously [70]. Briefly, 500 ng of integrin αvβ8 (R&D System, Cat# 4135-AV-050), fibronectin (Thermo, Cat# PHE0023), and humanin and scrambled humanin (Anygen, Cat# AGP-8245) were blotted onto nitrocellulose membranes (BioTrace, Cat# 66485). The membranes were then blocked with 3% BSA in TBST, followed by the incubation with either scrambled humanin, humanin, or integrin αVβ8 solution (5 µg/mL) overnight at 4 °C. Membranes were then washed three times with TBST before incubating with anti-integrin αV (CST, Cat# 4711 S), anti-integrin β8 (SCBT, Cat# sc-514150) or anti-humanin (Neuromics, Cat# RA19000) antibodies overnight at 4 °C. After washing with TBST; the membranes were incubated with anti-rabbit IgG (Cat # G21234; Invitrogen) or anti-mouse IgG (Cat# A16066) as a secondary antibody for 1 h at room temperature, followed by detection using an ECL solution (Cat # RPN2235; GE Healthcare).

### 3D invasion assay

The protocol for this assay was adapted from a previous study [71]. In brief, 83 cells were seeded into a 96-wells plate at a concentration of 5 × 10^4^/mL and incubated at 37 °C, 5% CO_2_ atmosphere condition. After 2 days, the Cultrex Basement Membrane Extract (R&D, Cat# 3632-010-02), mixed with either scrambled humanin or humanin, was added to each well using a cold tip. For live-cell visualization, the cells were then stained with DAPI nuclear-staining reagent (Abcam, Cat# ab228549) before acquiring pictures. A series of pictures were taken at the beginning (T0) and after 48 h (T48) after the introduction of the basement membrane. The level of cell invasion was determined by the changes in the cell spreading area, calculated using ImageJ software.

### Survival analysis using the Chinese Glioma Genome Atlas (CGGA) dataset

The analysis of raw data from the CGGA dataset (cgga.org.cn) was carried out using the GlioVis website (http://gliovis.bioinfo.cnio.es/), as reported previously [72]. Briefly, only the GBM patient data were selected for analysis. The cutoff value was determined using the maximally selected rank statistics for continuous variables, as provided in the “survminer” R package, which was also integrated into GlioVis platform. Raw data downloaded from the website was visualized using GraphPad Prism 8.

### Genes Expression Atlas Analysis using CCLE dataset

The analysis was performed using the Expression Atlas website (https://www.ebi.ac.uk/gxa/home), which has been reported previously [73]. Briefly, the expression of human integrin αV, integrin β6, and integrin β8 in SCLCs and glioblastoma (CCLE dataset) was selected for further analysis. Raw transcript per million (TPM) values were downloaded and visualized using GraphPad Prism 8.

### Relative RT-PCR analysis

Total RNA was extracted from cells using TRIzol reagent (Invitrogen, Cat# 15596018) following the manufacturer’s instructions. cDNA was reverse-synthesized using the qPCR RT Master Mix (Toyobo, Osaka, Japan, Cat# FSQ-301). The mRNA expression of the genes of interest was determined through real-time PCR (RT-PCR) using the ABI Prism 7900 HT Sequence Detection System (Applied Biosystems). Three replicates of each PCR reaction were performed using SYBR Green RT-PCR master mixes (Life Technologies, Cat# 4309155). Data were analyzed using the delta-delta Ct method with 18S rRNA as the reference gene. Primer sequences are listed in Supplementary Table 1.

### Integrin αV knockdown using siRNA

For siRNA transfection, X02 and 83 were seeded at 1.5 × 10^5^ cells per well. Cells were transfected with control siRNA or siRNA targeting integrin αV at a final concentration of 100 nM for 1 d using the reverse transfection method with Lipofectamine 2000 reagent (Thermo Fisher Scientific, Cat# 11668-019), following the manufacturer’s instructions. The cells were then collected for further analysis using immunoblotting and cell attachment assays. The siRNA sequences are shown in Supplementary Table 2.

### Cell migration assay

Cell migration assay was performed using a cell migration kit (Cat# K906-100) obtained from BioVision (Milpitas, CA, USA). GSCs, including X02, 83, and 1123 were seeded at 50,000 cells per well in the top chamber. After 1 d, humanin was added to the bottom chamber alongside the positive control, while inhibitors or antibodies were added to the top chamber. After 24 h, cells were washed and incubated in the dye for 2 h at 37 °C, followed by changes in fluorescence intensity at Ex 530 nm/Em 590 nm using a Flex station (Molecular Devices, CA, USA) for quantification. The number of cells was then calculated using a standard curve, as instructed by the manufacturer.

### Live-cell imaging

Prior to the experiments, X02 cells were coated onto a FlouroDish (World Precision Instrument, Cat# FD35-100) using Cell-tak (Corning, Cat# 354230) at the density of 3 × 10^5^ cells per well. The next day, cells were then treated with Scrambled humanin, humanin, TGFβ, GLPG0187 or anti-TGFβ and recorded using 3D Cell Explorer-fluo machine (Nanolive Inc., Switzerland) for 24 h. For the quantification, time-lapse videos were exported at a rate of 60 fps, with a total of 2500 frames per video. These videos were then imported into ImageJ and analyzed using Manual Tracking plug-in. Each cell was tracked from the beginning with the increment of 200 frames until the 2000th frame. Subsequently, the relative travel distance and velocity were calculated and visualized using GraphPad Prism 8.

### Rac1/Cdc42 activity assay

The activity of Rac1/Cdc42 was measured using the kit from Cytoskeleton Inc. (Cat# BK030) following the manufacturer manual. After the pulldown, the samples were then subjected to western blotting against Rac1/Cdc2 antibody (provided in the kit). Horseradish peroxidase HRP-conjugated anti-mouse IgG (Invitrogen, Cat# A16066) was used as the secondary antibodies. The membranes were detected using an ECL solution (GE Healthcare, Cat# RPN2235) and visualized by the Bio-Rad ChemiDOC XRS+ system.

### Statistical analysis

All data are reported as mean ± SEM (*n* = 3, except indicated). All graphs and statistical comparisons were performed using GraphPad Prism 8 with the Student’s *t*-test and one-way ANOVA. A *P*-value less than 0.05 was considered statistically significant.

### Supplementary information

Supplementary Materials

Scramble Humanin

Humanin

Humanin + TGFβ

Humanin + GLPG0187

Humanin + TGFβ

Original WB

## Data Availability

This study deposits no data in external repositories.
