# Supplementary material for: Humanin activates integrin αV–TGFβ axis and leads to glioblastoma progression
Source: Cell Death Dis. 2024 Jun 28;15(6):464. doi: 10.1038/s41419-024-06790-8 (PMC11213926; doi:10.1038/s41419-024-06790-8)
Supplement: Supplementary file 1 — Supplementary Materials [file 41419_2024_6790_MOESM1_ESM.pdf]

Supplementary information

"Humanin Activates Integrin αV–TGFβ axis and leads to Glioblastoma Progression"

Cuong P. Ha, Tuyen N.M. Hua, Vu. T.A. Vo, Jiyeon Om, Sangwon Han, Seung-Kuy Cha, Kyu-Sang Park and Yangsik Jeong

Supplementary Figures

Supplementary Figure 1. **High expression of *MT-RNR2* transcripts in the brain compared to other tissues.** (**a**) Relative expression of *MT-RNR2* was normalized by *ATP6* using GTEx portal resources. (**b**) Heatmap analysis was executed for nucleus-encoded humanin-like genes in multiple tissues of healthy donors using the GTEx portal resource.

c, d Humanin expression is higher in the GBM tumor tissue compared to the pair-matched normal tissue. Using eight pair-matched tissues from the GBM patients, IHCs were performed for the expression of humanin peptide (**c**), followed by quantification (**d**). Each dot in the quantification graphs represents an individual staining region. Statistical analysis was performed using a 2-tailed Student's t-test. *$P < 0.05$, **$P < 0.01$, ***$P < 0.001$, ****$P < 0.0001$. Scale bar 100 μm.

Supplementary Figure 2. **Humanin treatment leads to GSC attachment.** (**a**) GSCs were treated with 20 μM of humanin in a time-dependent manner followed by observation of morphological changes using a brightfield microscope or quantification of the cell attachment assay (right). (**b**) X02 cells were treated with humanin or HL8/HL12 alone or a mixture of the two peptides with different ratios for 24 h, followed by observation of morphological changes using a brightfield microscope (left) with cell attachment quantification (right). (**c**) SCLCs, including H69, H82, and H889 cells, were treated with 20 μM of humanin for 24 h, followed by morphology observation using a brightfield microscope. (**d**) Expression of genes of interest associated with cellular attachment upon humanin treatment. Statistical analysis was performed using one-way ANOVA (**a, b**) or two-tailed Student's *t*-test (**d**). *$P < 0.05$, **$P < 0.01$, ***$P < 0.001$, ****$P < 0.0001$. Scale bar 250 μm for images.

Supplementary Figure 3. **Humanin-induced attachment requires integrin αV.** (**a**) Relative expression of humanin receptors and binding partners. Using qPCR assay, the mRNA

expression of known humanin receptors, *WSX1*, *GP130*, *CNTFR*, and *FPRL1*, and binding partners, *IGFBP3* and *BCL2*, was determined in the panels of GSCs and SCLCs. (**b, c**) Intracellular STAT3 and ERK signaling for the known humanin receptors were evaluated in the same panel of GSCs and SCLCs, basally (**b**) or upon 20 μM of humanin treatment for 24 h (**c**) using immunoblot assay. (**d**) X02 cells were treated with 20 μM of humanin or $MgSO_4$ and $CaCl_2$ at indicated doses, followed by observation of morphological changes using a brightfield microscope (left) and quantification of cell attachment assay (right). (**e-g**) Integrin signaling and extracellular calcium are critical for humanin-induced GSC attachment. X02 cells were treated with 20 μM of humanin followed by 10 μM GLPG 0187 (**e**), 2 mM of extracellular calcium chelator EGTA (**e**), or 2 or 5 μM intracellular calcium chelator EGTA-AM (**f**) for 24 h. (**f**) Morphological change (left) or quantification of cell attachment (right) is represented. (**g**) Immunoblot assay for intracellular signaling of integrin or TGFβ receptor. (**h**) Integrin αV and β8 expressions are significantly higher in GSCs than SCLCs. Using the public CCLE dataset, the TPM of integrin αV, β6, and β8 were analyzed in GSCs and SCLCs. (**i**) Humanin binding to integrin αV. 83 GSCs cells were treated with 20 μM humanin for 24 h before collecting lysates for co-immunoprecipitation experiments in which humanin was immunoblotted after immunoprecipitation of integrin αV.

j 83 cells were treated with 20 μM of scrambled humanin or humanin for 24 h, with or without integrin αV knockdown, followed by immunoblot assay (left), cell attachment (middle), or morphological observation using a brightfield microscope (right).

Data information: Statistical analysis was performed using one-way ANOVA (**d, f, j**) or two-tailed Student's *t*-test (**h**). ****$P < 0.0001$. Scale bar 250 μm for images.

Supplementary Figure 4. **Humanin-induced attachment is independent of the Rho-ROCK signaling pathway.** X02 cells were treated with 20 μM of humanin or Rho/ROCK

inhibitor Y27632 in a dose-dependent manner for 24 h, followed by observation of morphological changes, using a brightfield microscope. Scale bar 250 μm for images.

Supplementary Figure 5. **Humanin activates TGFβ signaling pathway.** (**a**) GSCs cells were treated with 20 μM of humanin for 24 h (upper) or in a time-dependent manner (lower), followed by immunoblot assay. (**b**) Evaluation of intracellular TGFβ signaling upon humanin treatment. X02 and 1123 cells were treated with 20 μM of humanin in combination with 1 μg/mL anti-TGFβ antibody for 24 h, followed by immunoblot assay for proteins of interest. (**c**) No response of TGFβ signaling to humanin treatment in SCLC. H82 cells were treated with 20 μM of humanin for 24 h, followed by observation of morphological changes using a brightfield microscope (left) and immunoblot assay for pSmad2 activation (right). (**d**) Assessment of TGFβ signaling upon inhibition of the humanin-activated integrin signaling. X02 cells were treated with integrin inhibitors, including various doses of cilengitide (10 µM and 20 µM) or GLPG0187 (1–50 nM) in the presence or absence of 20 µM humanin for 24 h, followed by immunoblot assay. G, GLPG0187; C, cilengitide. (**e**) The humanin-induced attachment of GSCs is not affected by modulating TGFβ signaling. X02, 83, and 1123 cells were treated with 20 μM of humanin in the presence of TGFβ signaling pathway inhibitors, including 1 μg/mL anti-TGFβ antibody (left) and 10 μM of SD208 (right) for 24 h, followed by morphological observation. (**f**) Identification of TGFβ receptor signaling in the GSCs. X02, 83, and 1123 cells were treated with 0.5 ng/mL of TGFβ, 10 μM of SD108, or in combination for 24 h, followed by immunoblot assay (left) and observation of morphological changes by bright field microscope (right). Scale bar 250 μm for images. Scale bar 250 µm for images..

Supplementary Figure 6. **Humanin elicits cell migration of the GSCs**.

a Evaluation of cell migration in various inhibitors condition. 83 cells were treated with 20 μM of humanin in the presence of 2 mM EGTA, 10 μM GLPG0187 for integrins inhibitors, 5 μM

Wiskostatin for N-WASP inhibitor or 50 nM MBQ167 for Rac/CDC42 inhibitor, 0.5 ng/mL TGFβ, 1 μg/mL anti-TGFβ, or 10 μM SD208 for TGFβ receptor for 24 h, or 5 μg/mL anti-humanin antibody for 24 h, followed by cell migration assay.

b Invasiveness of 83 cells was assayed upon humanin or scrambled humanin treatment with DAPI staining. The invasion was represented in the picture (left) with quantification by using ImageJ software (right).

c-d Quantification of live cell imaging for relative velocity (**b**) and travel distance (**c**) for supplementary video 1. X02 was treated with 20 μM scrambled humanin or humanin, 10 μM GLPG0187, 0.5 ng/mL TGFβ or 1 μg/mL anti-TGFβ for 24 h.

e Representative images for the H&E staining for tumor visualization of the orthotopic xenograft tumor model in both scrambled humanin (n=3) and humanin (n=7) group.

f Immunofluorescence staining for CD31 expression in orthotopic xenograft tumor model with representative images (left) and quantification (right).

Data information: In (**a, c, d**), Statistical analysis was performed using one-way ANOVA. In (**b, f**), statistical analysis was performed using two-tailed Student's *t*-test. *$P < 0.05$, **$P < 0.01$, ***$P < 0.001$, ****$P < 0.0001$.

Supplementary Figure 1

a

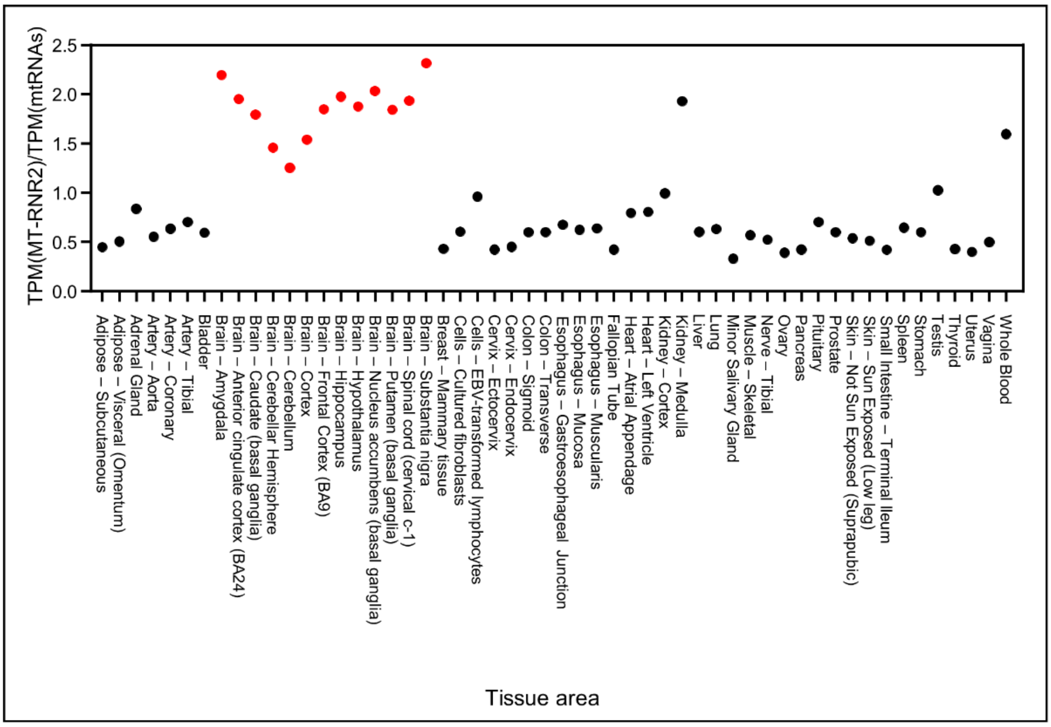

b

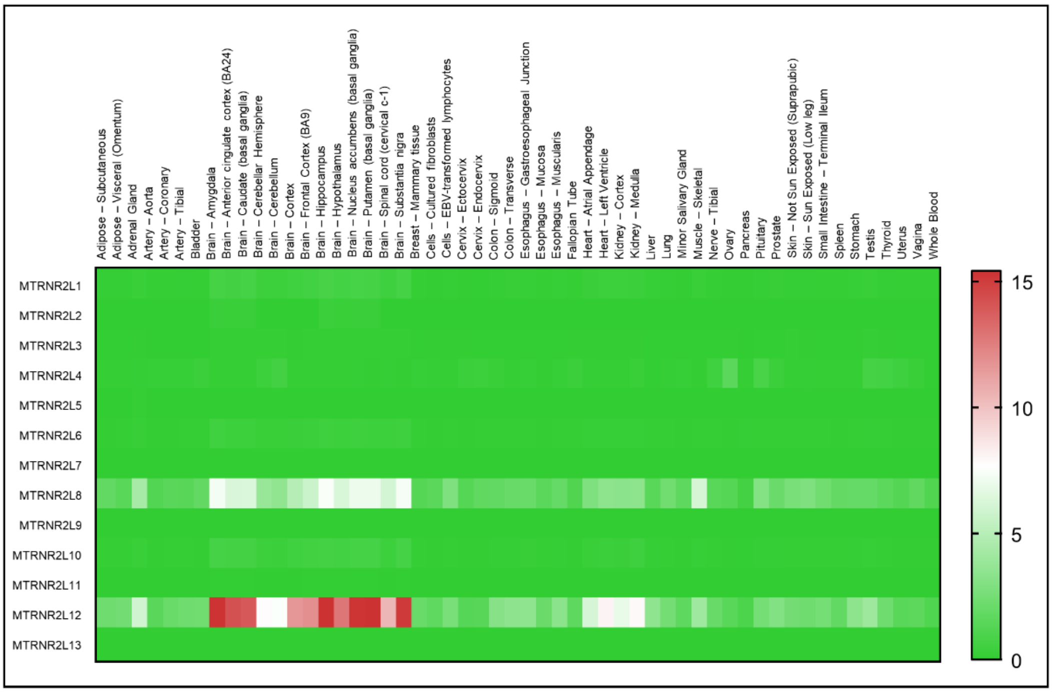

Supplementary Figure 1

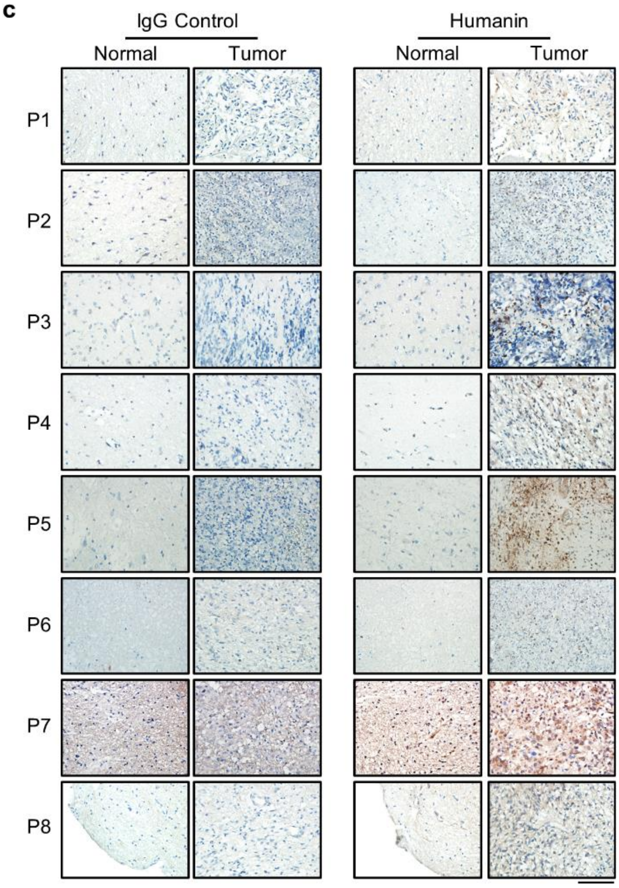

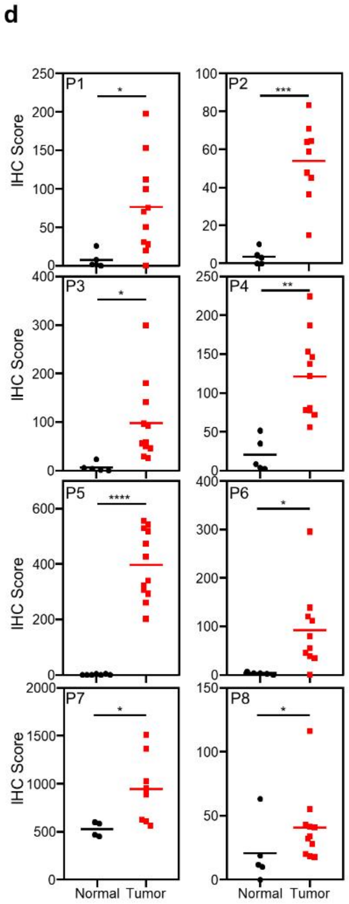

Supplementary Figure 2

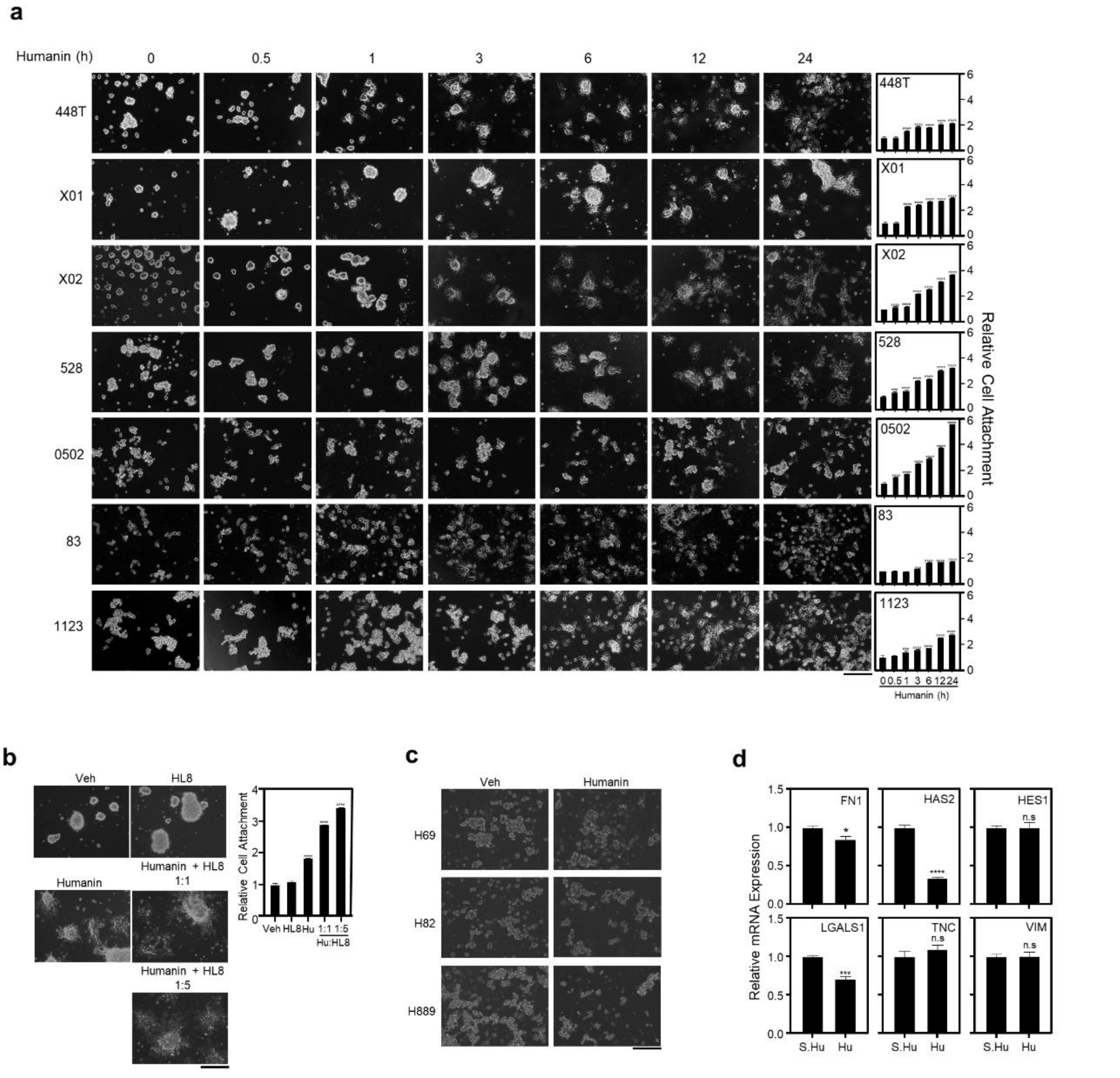

Supplementary Figure 3

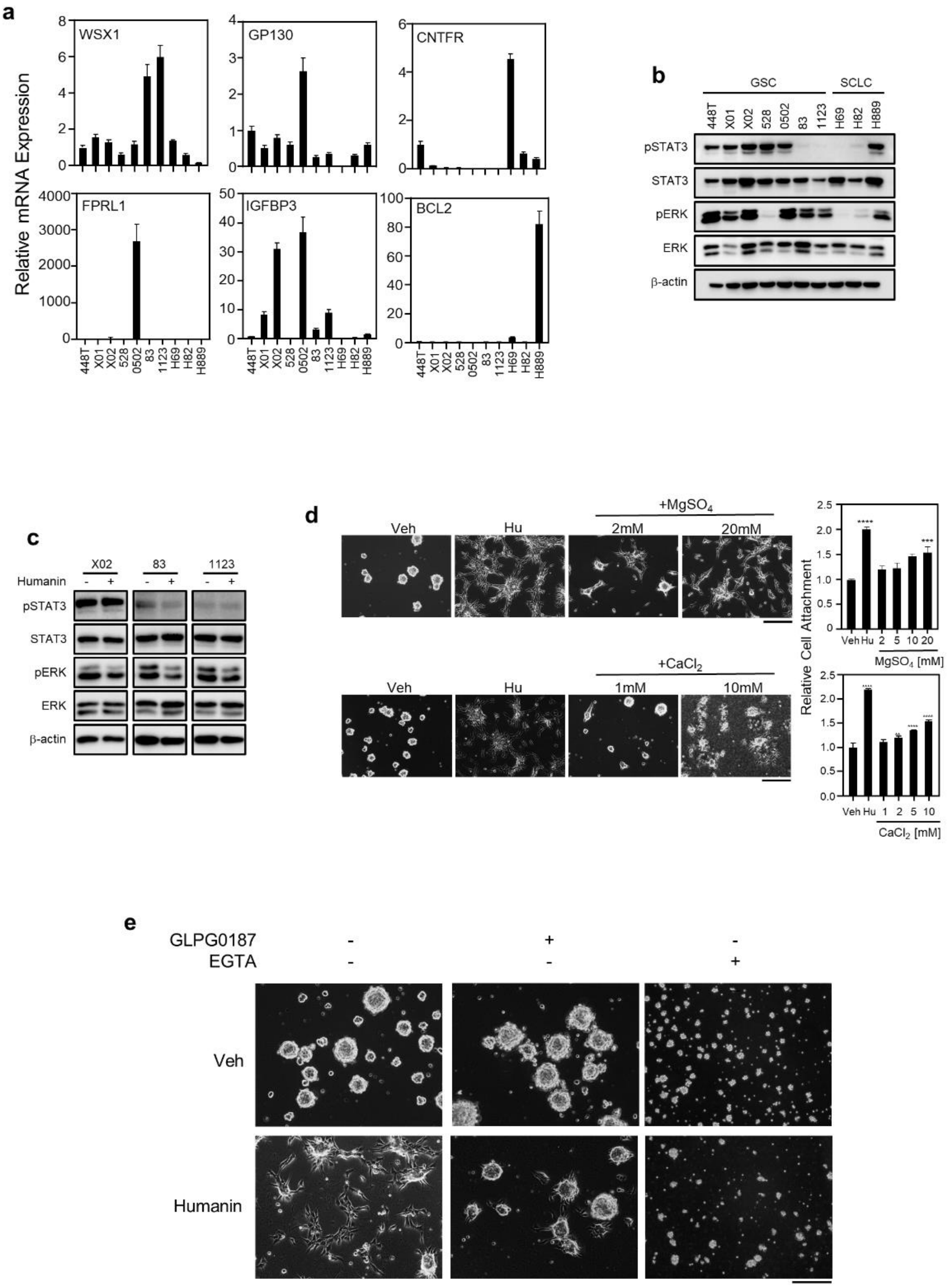

Supplementary Figure 3

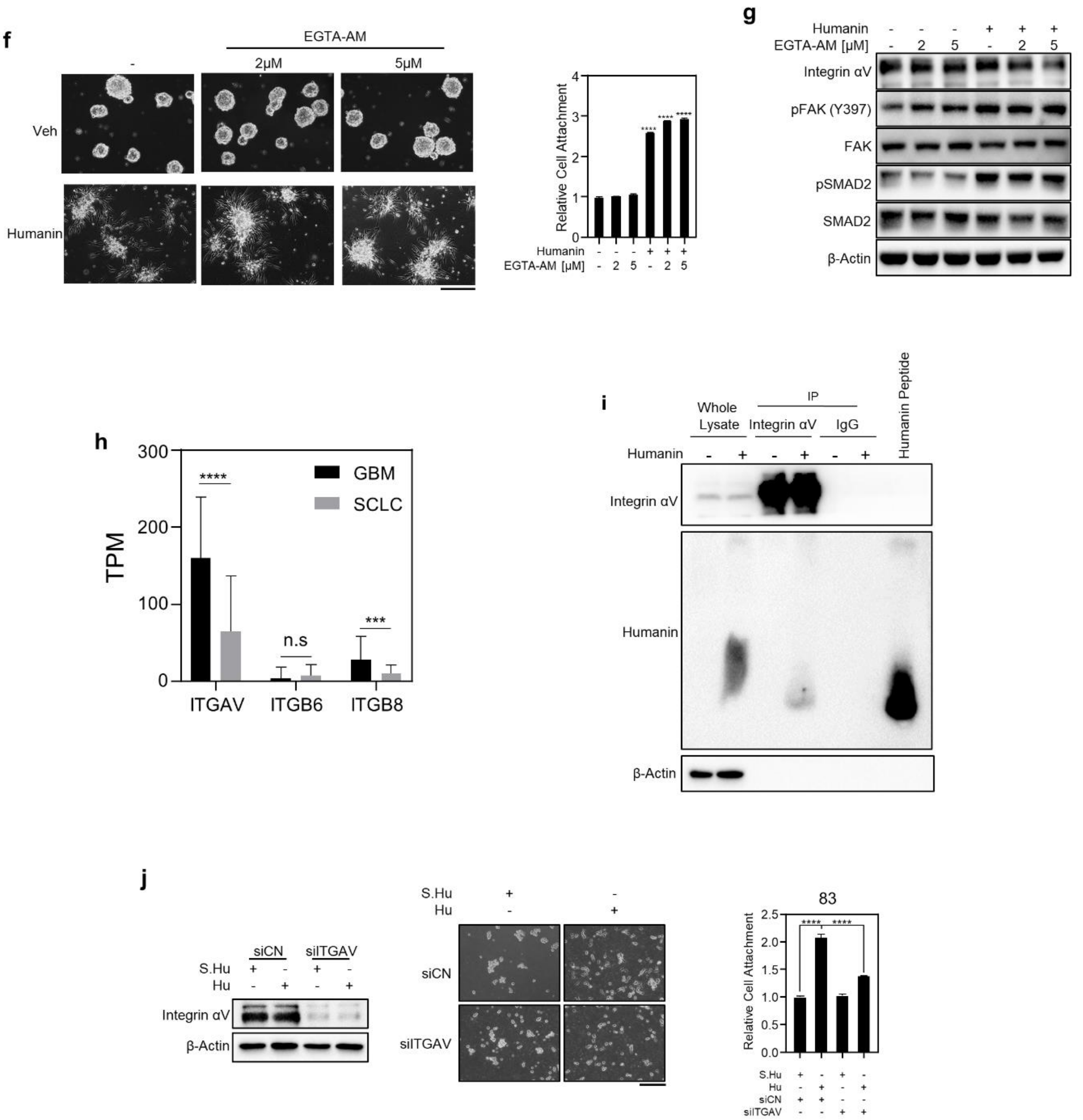

Supplementary Figure 4

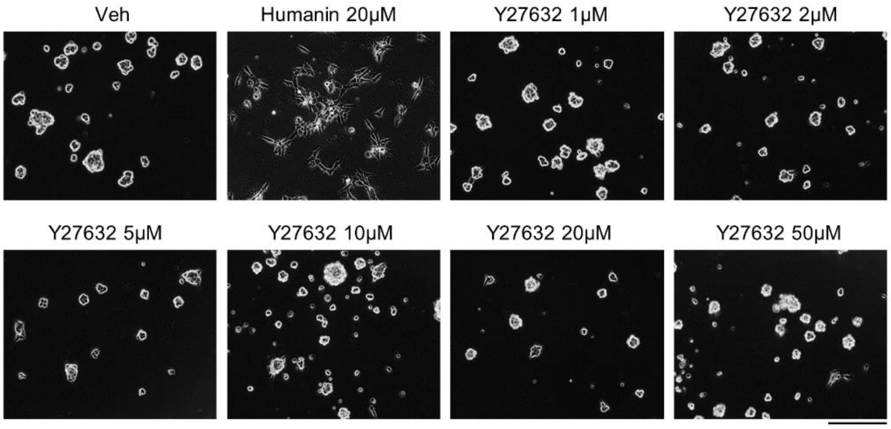

Supplementary Figure 5

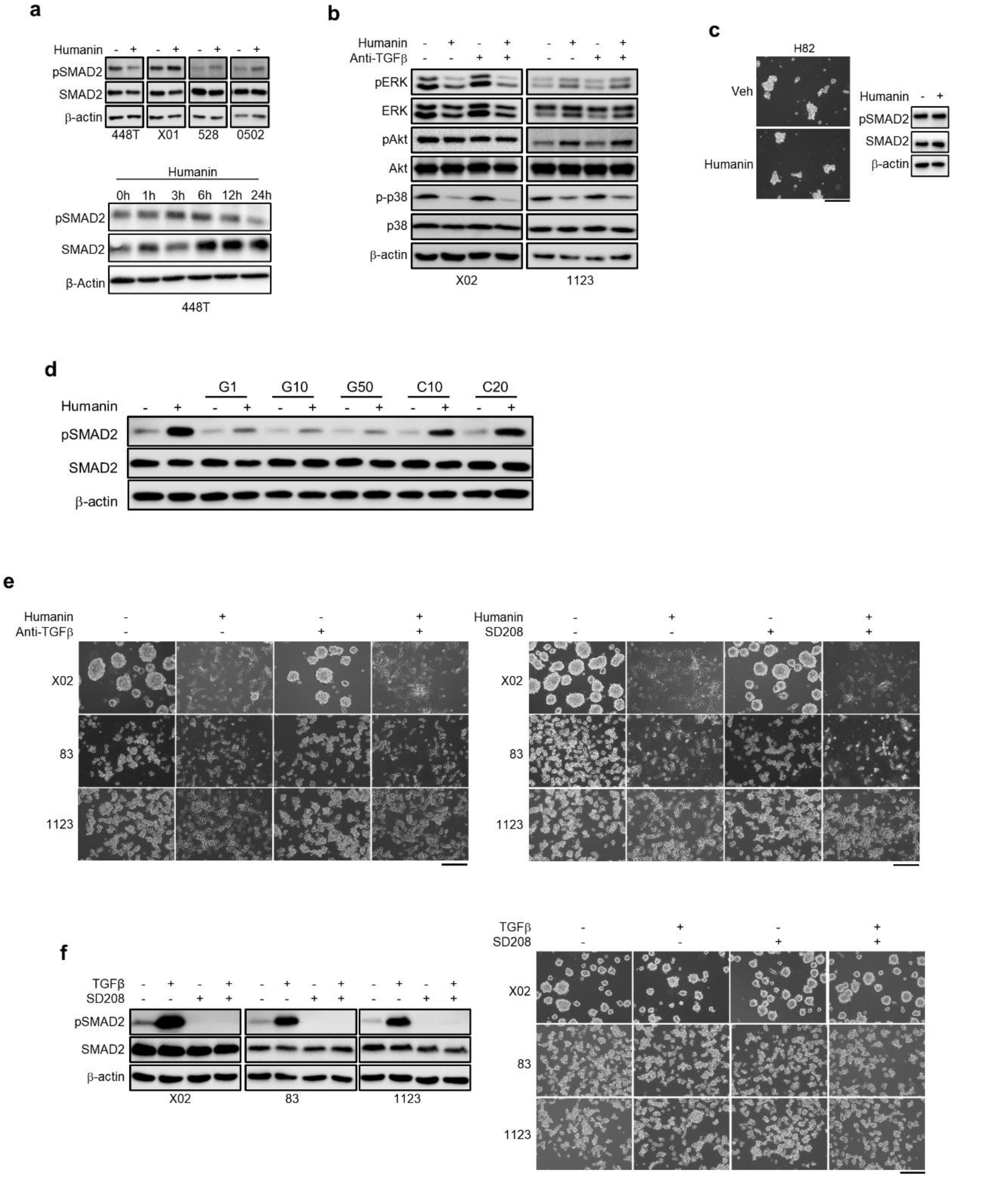

Supplementary Figure 6

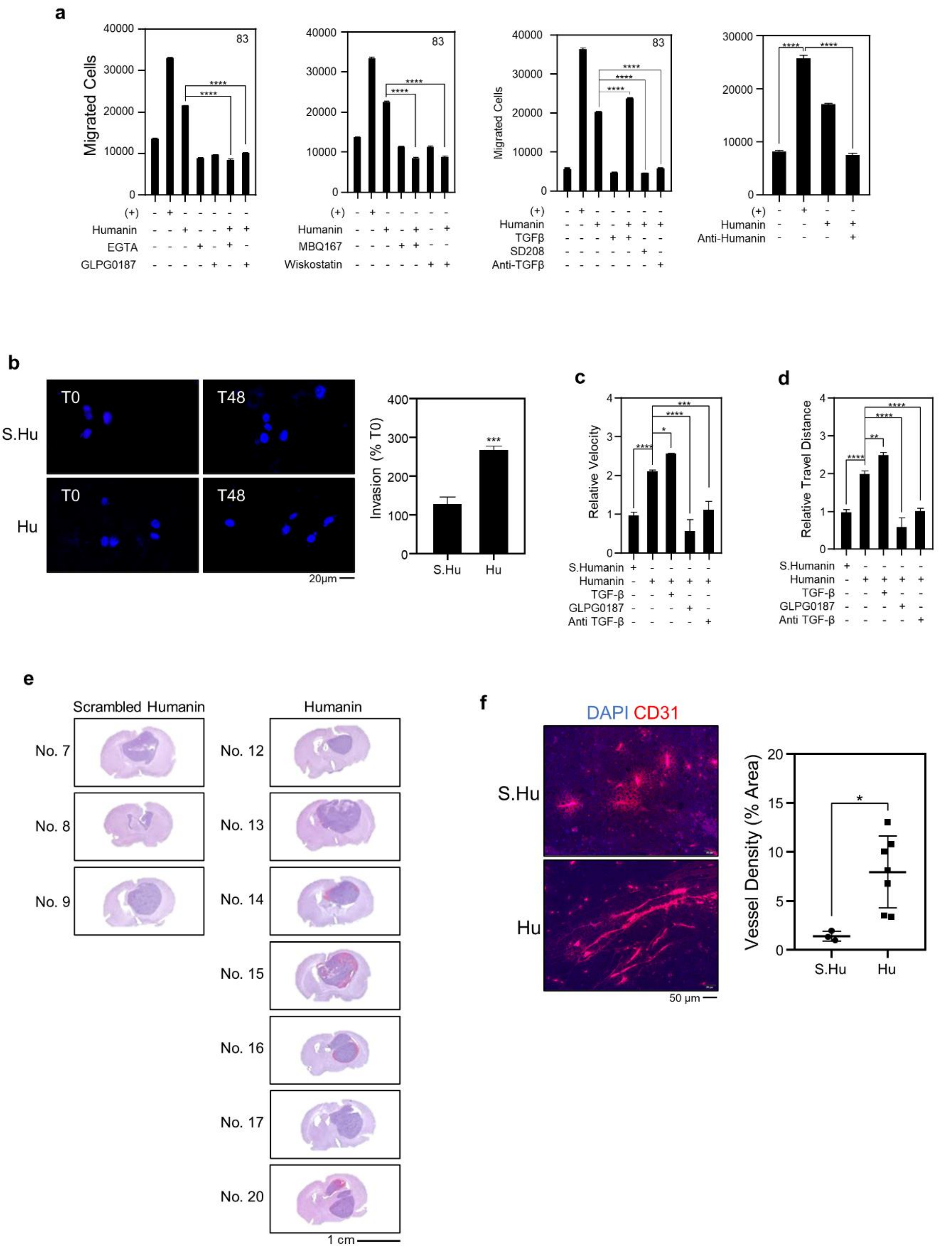

Supplementary Video

Supplementary video 1: Live-cell imaging of X02 under the treatment of 20 μM scrambled humanin or humanin, 10 μM GLPG0187, 0.5 ng/mL TGFβ or 1 μg/mL anti-TGFβ for 24 h.

Supplementary Table 1. Primer sequences for RT-PCR.

**Gene name**	**Sequence (5′-3′)**
18S	Forward: ACCGCAGCTAGGAATAATGGA<br>Reverse: GCCTCAGTTCCGAAAACCA
WSX1	Forward: GAGCCCCCTCCGAGTTACAC<br>Reverse: AGCTGTTCCCGAGGAATGG
FPRL1	Forward: TCCCTGGCCTTCTTCAACAG<br>Reverse: AGCCGTGTCATTAGTTGGGG
CNTFR	Forward: GCCACATTCGCTACATGCAC<br>Reverse: TGGAGGATCAGGCTTCACAATG
GP130	Forward: TCAGGCTTGCCTCCAGAAAA<br>Reverse: CCTTCCACCATCCCACTCAC
IGFBP3	Forward: TCAATGTGCTGAGTCCCAGG<br>Reverse: AGGGCGACACTGCTTTTTCT
BCL2	Forward: TGGAGGAGCTCTTCAGGGAC<br>Reverse: CTCTCCACACACATGACCCC
FN1	Forward: CAGCCATCTCACATTTCCAA<br>Reverse: CGTACTGCTGGATGCTGATG
HAS2	Forward: GCCTCATCTGTGGAGATGGT<br>Reverse: GGAATGAGATCCAGGAATCG
HES1	Forward: TGAGCCAGCTGAAAACACTG<br>Reverse: CCGCGAGCTATCTTCTTCA
LGALS1	Forward: CTGTCTTTCCCTTCCAGCCT<br>Reverse: CTGGTCGAAGGTGATGCAC
TNC	Forward: TCTCAGGGTCATTCACCACA<br>Reverse: CACCGTGCGTGTAATTTCTG
VIM	Forward: AGGTGGACCAGCTAACCAAC<br>Reverse: ATTCCACTTTGCGTTCAAGG

Supplementary table 2. siRNA sequence of ITGAV.

siRNA	Sequence (5′-3′)
ITGAV siRNA 1 Target Sequence	GGAUGAAUCUGAAUUUAGA
ITGAV siRNA 2 Target Sequence	CCAUGUAGAUCACAAGAUA
ITGAV siRNA 3 Target Sequence	CGACAAAGCUGAAUGGAUU
ITGAV siRNA 4 Target Sequence	CCGAAACAAUGAAGCCUUA
