# Supplementary figures and images for: Humanin activates integrin αV–TGFβ axis and leads to glioblastoma progression
Source: Cell Death Dis. 2024 Jun 28;15(6):464. doi: 10.1038/s41419-024-06790-8 (PMC11213926; doi:10.1038/s41419-024-06790-8)

**a**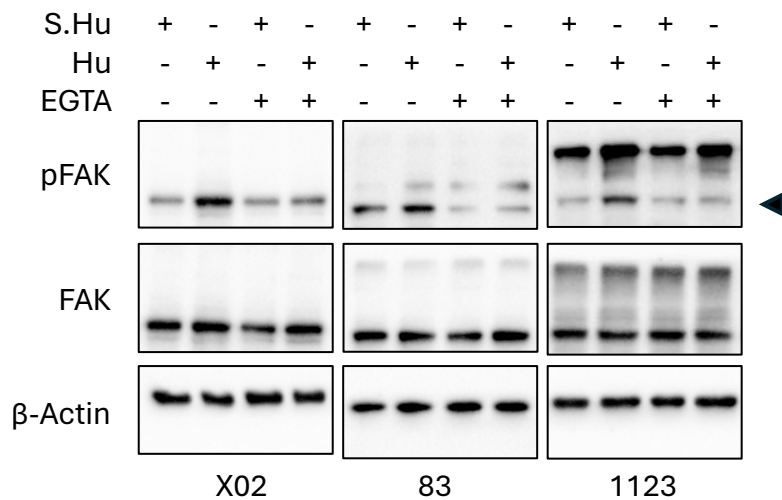**b**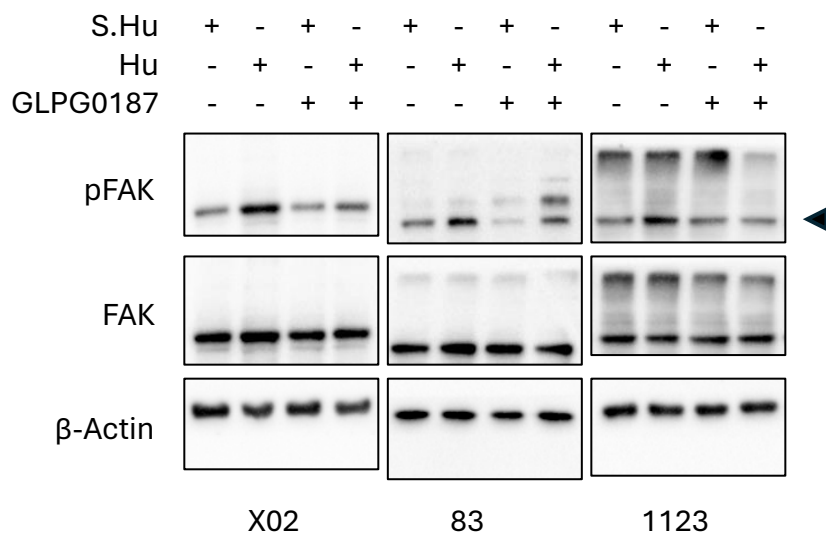**c**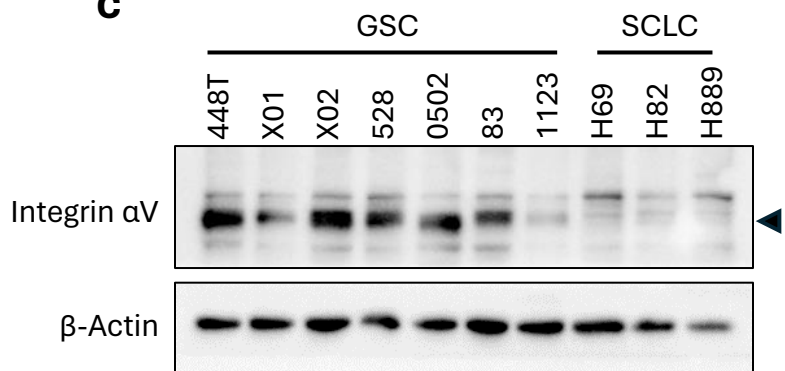**e**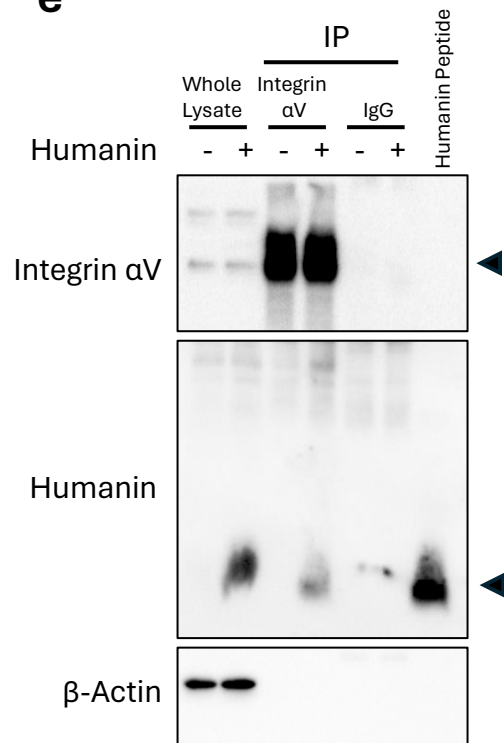**g**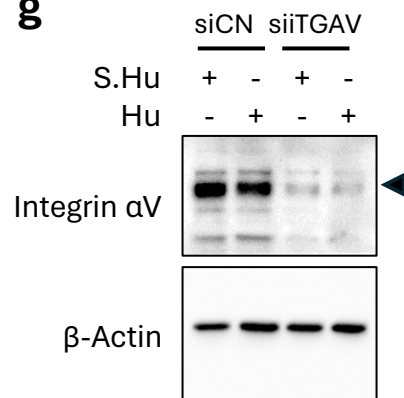

**d**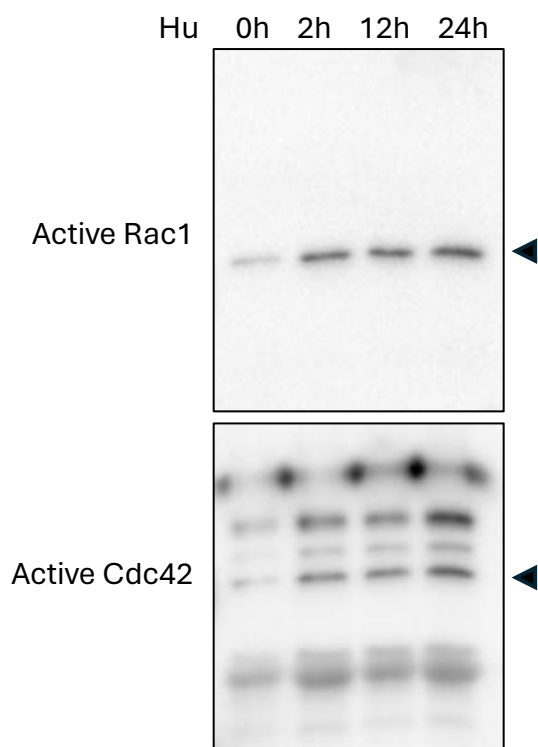

**a**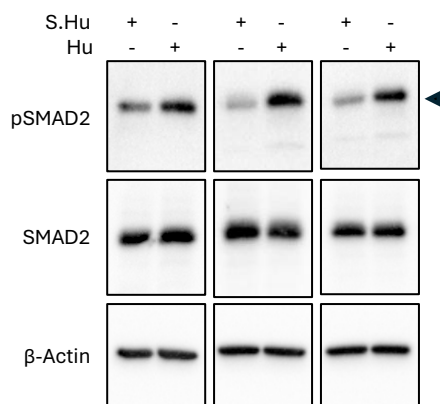**b**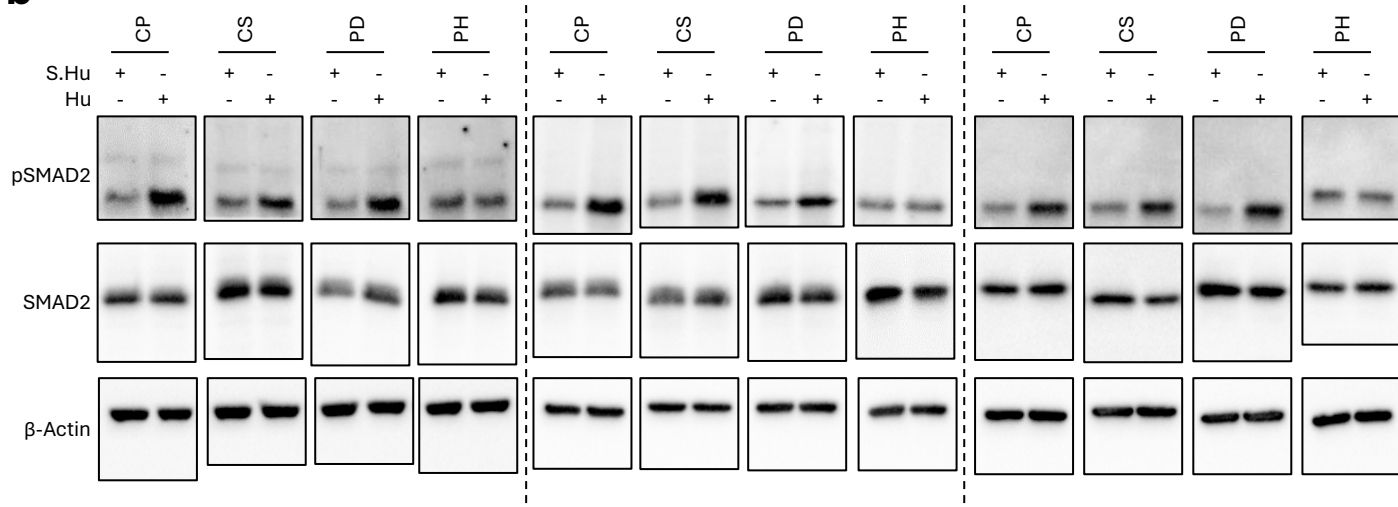**c**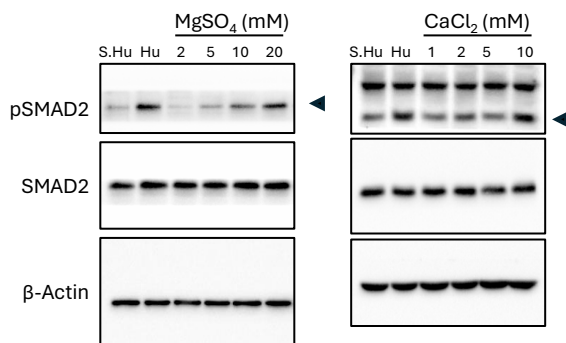**d**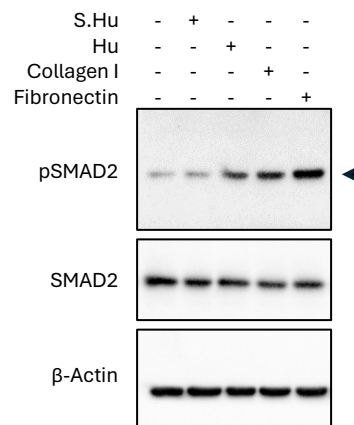

**e**

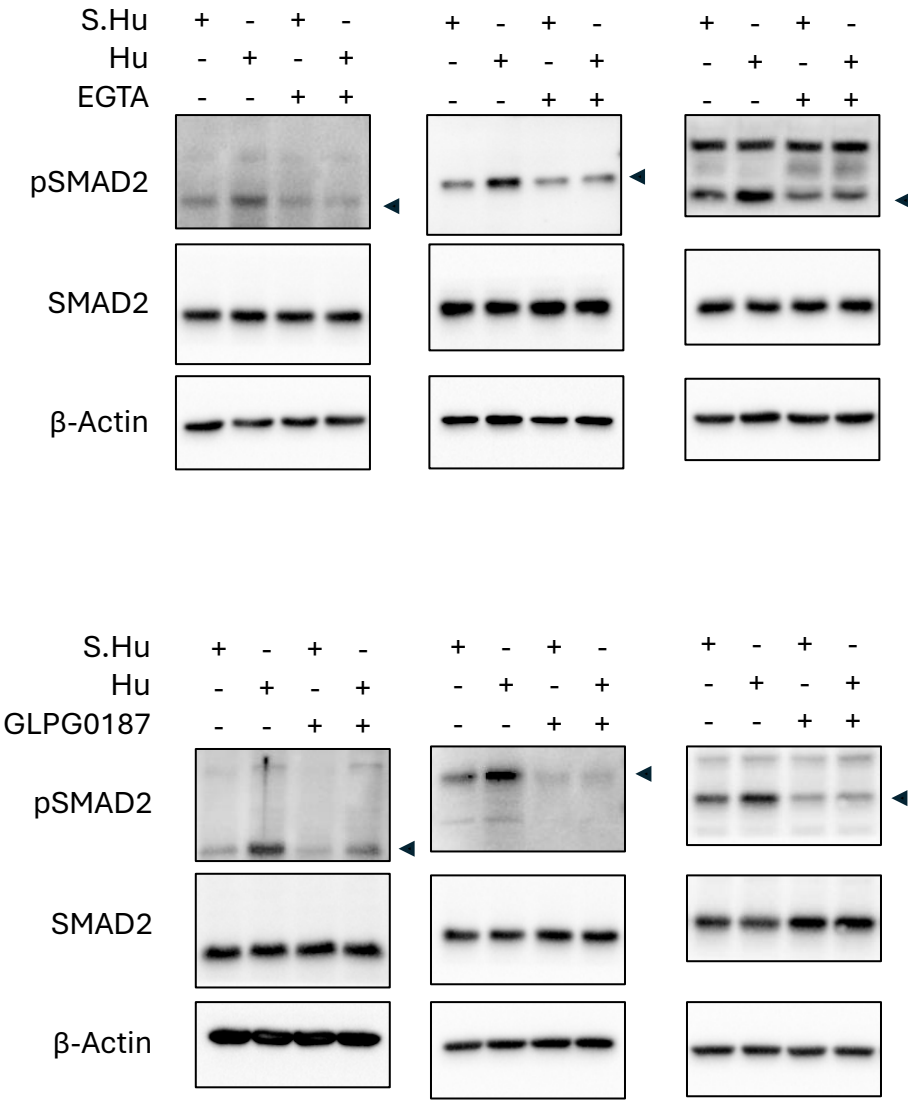

**f**

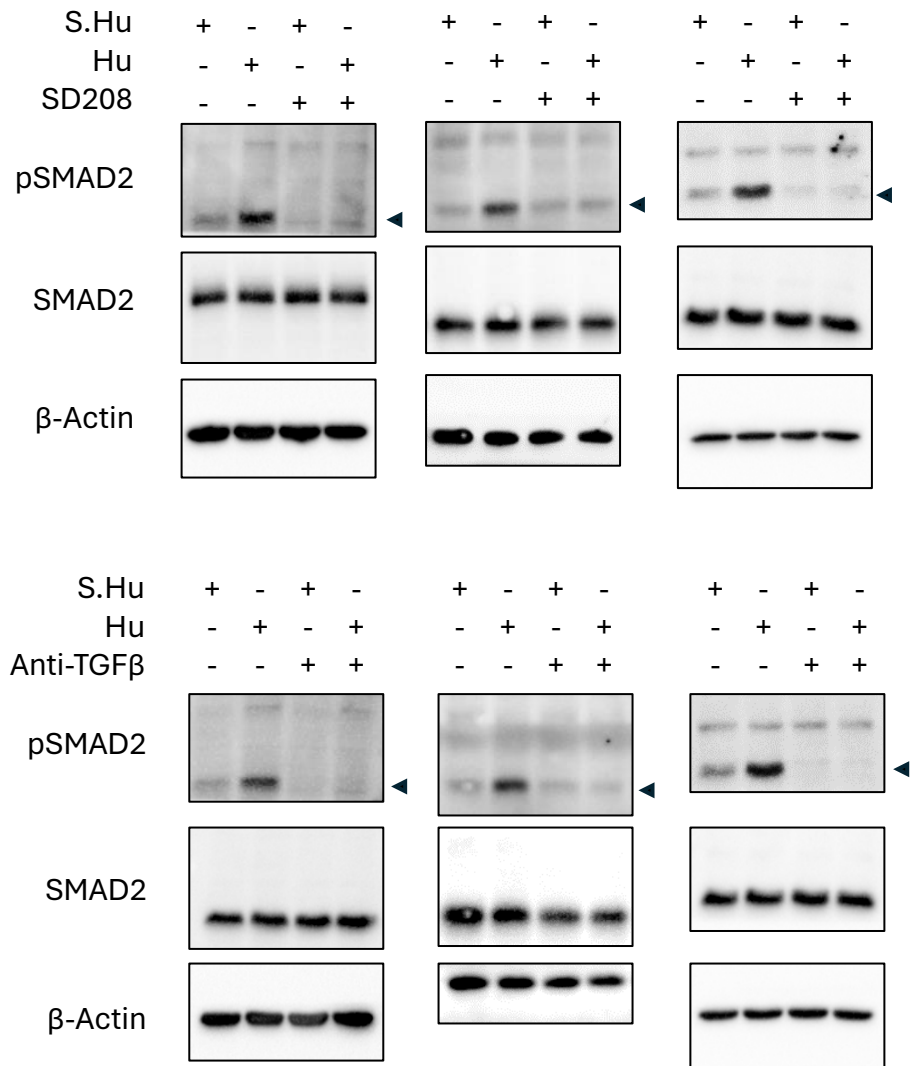

**h**

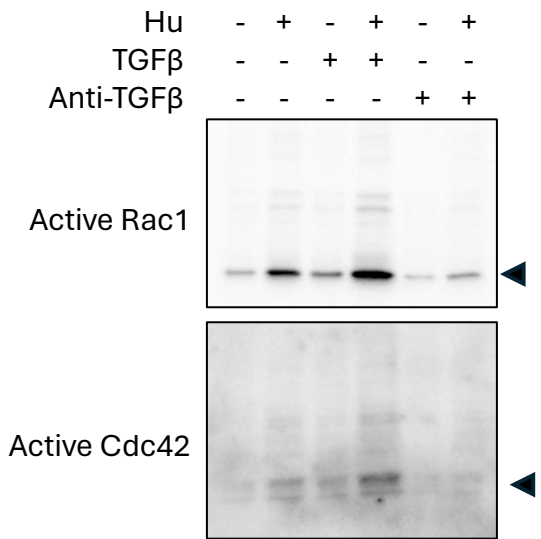

Supplement: Supplementary file 7 — Original WB [file 41419_2024_6790_MOESM7_ESM.pdf]
